# Integrated learning-assisted design of metal–nitrogen–carbon single-atom catalysts: electronegativity regulates the coupling rules of interfacial valence electrons

**DOI:** 10.1039/d6sc00422a

**Published:** 2026-05-05

**Authors:** Hang Zhang, Zishan Luo, Xi Sun, Yuhang Zhou, Wenhao Yan, Jiawei Li, Tao Zhang, Hong Cui, Weizhi Tian, Rong Feng, Hongkuan Yuan

**Affiliations:** a School of Mechanical Engineering, Shaanxi University of Technology Hanzhong Shaanxi 723001 China hongcui@snut.edu.cn tianweizhi@snut.edu.cn; b Shaanxi Key Laboratory of Industrial Automation, Shaanxi University of Technology Hanzhong Shaanxi 723001 China; c School of Physical Science and Technology, Southwest University Chongqing 400715 China

## Abstract

Single-atom catalysts (SACs) have demonstrated great potential in the electrochemical nitrogen reduction reaction (NRR). However, the electronic regulation mechanism of intermediate adsorption on SACs remains unclear, and conventional density functional theory (DFT) calculations fail to establish a universal “structure–performance” relationship. This study is based on coordinated single-atom structures (M-NnCm-GN), anchored at defect sites of nitrogen-doped graphene (GN), and develops an AdaBoost-XGB integrated model (*R*^2^ = 0.95) to analyze the interaction mechanisms between metal active sites and reaction intermediates (O, N, C and H). The results show that the doped metal in the MN4 structure and the adsorbed intermediates follow the 10-valence electron coupling rule, which is extended to different coordination environments. For the N intermediate, average electronegativity values less than 2.8, equal to 2.8 and greater than 2.8 correspond to the 11-, 10- and 10-valence electron coupling rules, respectively, while O follows the 10-valence electron coupling rule. In addition, we used this rule to guide the design of NRR (N_2_ → NNH) and OER (OH → O) catalysts. The three-dimensional descriptor of the adsorbate fitted by the SISSO algorithm achieved an *R*^2^ of 0.97, further improving predictive accuracy. The metal valence electron number (Ne_1_) exhibits a positive correlation with adsorption energy, while the introduction of bond length features (*d*_1_) enhances the model's predictive accuracy by approximately 17%. The electronegativity-regulated interfacial valence electron coupling rules established in this study successfully unravels the “structure–activity relationship black box” challenge of SAC catalysts, providing a quantifiable and transferable approach for the design of high-performance SACs.

## Abstract 2

By integrating DFT with ensemble learning, this work establishes electronegativity-regulated valence-electron matching rules for M-NnCm-GN SACs, where 10/11-electron coupling controls adsorption energy and guides NRR/OER catalyst design.

## Graphical abstract

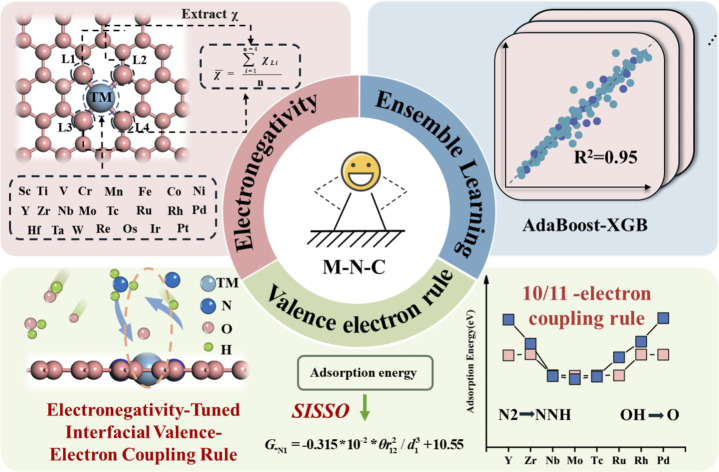

## 1 Introduction

Owing to their excellent activity and selectivity, single-atom catalysts (SACs) have become a focal point in energy conversion and engineering applications.^1–4^ They typically exist as isolated or atomically dispersed atoms, and their unique single active sites contribute to enhanced atomic utilization and catalytic selectivity.^5–7^ Zhu *et al.* constructed single-atom catalysts (SACs) on substrates such as graphene, MXenes, and transition metal sulfides through transition metal doping and have conducted extensive screening and predictive studies on their catalytic activities.^8^ The graphene-modified M-NnCm-GN structures (hereafter referred to as M–N–C) exhibit enhanced electronic coupling and structural stability^9–12^ and have been widely applied in catalytic reactions such as the oxygen reduction reaction (ORR), oxygen evolution reaction (OER),^13^ hydrogen evolution reaction (HER),^14^ nitrogen reduction reaction (NRR)^15^ and carbon dioxide reduction reaction (CRR).^16^ The M–N–C structure features a single transition metal (TM) atom covalently coordinated with the surrounding N/C atoms through four coordination bonds, thereby exhibiting unique structural characteristics.^17–19^ Chi *et al.* enhanced the performance of graphene-based single-atom catalysts by regulating the coordination environment of graphene.^20^ Although performance prediction based on first-principles calculations offers high accuracy, simple descriptors capable of capturing the overall trends of M–N–C systems are still lacking.^21–23^

For a wide range of reactions, the adsorption energetics of atomic and molecular intermediates on catalyst surfaces indicate the overarching activity tendency.^24–26^ According to the Sabatier principle, excessively strong adsorption energies of reaction intermediates hinder their desorption from the catalyst surface, whereas excessively weak adsorption energies prevent effective adsorption of the intermediates.^27,28^ The adsorption strength should remain moderate to achieve optimal catalytic activity,^29,30^ and the adsorption energy is determined by the electronic properties of the catalyst.^31^ In traditional catalytic systems, the position of the d-band center reflects the adsorption strength of intermediates and the catalytic performance.^32,33^ When the d-band center is closer to the Fermi level, the adsorption becomes stronger,^34,35^ whereas when it is farther from the Fermi level, the adsorption weakens,^36,37^ potentially reaching an ideal moderate range. Zhang *et al.* extensively applied the d-band center theory to catalyst model screening and achieved remarkable results in related studies.^38^ However, certain limitations still exist in catalyst systems such as graphene, MXenes and alloys.^39^ For example, in single-atom catalytic systems, fluctuations of the d-band center often need to be corrected to maintain consistency with the variation trends of target parameters such as adsorption energy or overpotential.^40–42^ Therefore, a concise and universal descriptor is urgently needed to describe the variation trends of adsorption energy.

Bai *et al.* successfully predicted adsorption energies and the overpotentials of the ORR and OER using traditional machine learning (ML) methods; however, these models suffer from poor interpretability and overly complex feature sets.^43^ In contrast, Lu *et al.* applied ensemble models that integrate the advantages of multiple algorithms, thereby improving prediction accuracy and exhibiting greater robustness and applicability across various catalytic reactions in complex systems.^44^ Song *et al.* used the SISSO algorithm to build concise descriptors for predicting adsorption energies on catalyst surfaces and revealed that the metal valence electron number (Ne) is the primary factor influencing adsorption energy variation.^45^ Meanwhile, the catalyst screening efficiency was improved by 2–4 orders of magnitude while retaining high prediction accuracy.^46^ Recently, a series of descriptors reflecting the intrinsic nature of catalytic activity have been developed based on electronic structure characteristics and geometric structural parameters. For example, Gao *et al.* constructed a general and interpretable descriptor based on electronegativity and d-electron count, which can efficiently predict the activity and selectivity of diatomic catalysts (DACs) in various C–C coupling reactions.^47^ Ren *et al.* proposed a single-atom saturation (SSA) principle, which evaluates catalytic activity in various reactions by combining the variations in the electronic structure (d-electron occupancy saturation) and geometric structure (coordination saturation) of individual guest atoms, together with the host atom type and intermediate adsorption configurations.^48^ However, these descriptors rely heavily on large amounts of DFT-calculated data and complex machine learning models, posing challenges for catalyst design.

Julia Schumann *et al.* proposed that the molecular research approach adopted by Julia Schumann and colleagues treats adsorbates uniformly as “atomic and molecular analogues”,^49^ in combination with orbital filling rules and partial valence electron coupling principles (such as the octet rule and the 4*n* + 2 rule).^50–52^ This method performs well in handling typical structures but encounters certain difficulties when the coordination environment varies.^53^ In-depth investigations into the electronegativity of the coordination environment and valence electron coupling rules are expected to overcome existing limitations and provide new design strategies for single-atom doping on supports such as graphene and MXenes.

Therefore, we have constructed and designed SACs from a completely new perspective. First, an integrated learning framework based on the DFT-EL approach was established. Using 210 DFT-calculated adsorption energies as target values, we constructed an AdaBoost-XGB ensemble model (*R*^2^ = 0.95). Feature importance analysis was performed to identify the main factors influencing the variation in adsorption energy, revealing that the metal valence electron number (Ne_1_) and the average electronegativity of the coordination environment (*χ*_6_) are the key factors. It reveals the matching mechanism between the metal valence electron number (VM) and the effective valence electron number (*k*) of the adsorbed intermediates (O/N/C/H) and the regulation mechanism of coordination environment electronegativity variations on the interfacial valence electron rules. The results show that when the electrons provided by the adsorbed intermediates (O/N/C/H) occupy and fill the d orbitals of the doped metal, the adsorption configuration becomes the most stable, thus leading to the proposal of the 10-valence electron coupling rule. In addition, this rule is used to derive the 11- and 10-valence electron coupling rules in different coordination environments. DFT calculations show that the S- and O-modified M–N–C structures also follow this rule, further supporting the universality of the law. This rule is also extended to the rate-determining steps (RDS) of both the NRR and OER, where it holds true. The electronegativity-regulated interfacial valence electron coupling rule proposed herein can serve as a physical descriptor to explain the adsorption energy variations induced by coordination environment effects. Subsequently, the electronic structures (atomic/molecular) of the M–N–C adsorbed intermediates were analyzed, revealing the mechanism by which the coordination environment regulates the interaction between the doped metal and the adsorbed intermediates. Finally, the SISSO algorithm was used to fit the adsorption energy data of (O, N, C and H)-M–N–C, constructing multiple simple three-dimensional descriptors for adsorption energy (with a maximum *R*^2^ = 0.98). In addition, the introduction of the distance feature (*d*_1_) between the doped metal and the adsorbed intermediates improves the model's predictive accuracy by approximately 17%. These simple descriptors can be directly used for adsorption energy prediction. This method eliminates the need for complex large-scale DFT calculations and can quickly provide guidance for the design and construction of four types of catalysts.

## 2 Methods

**Fig. 1:**
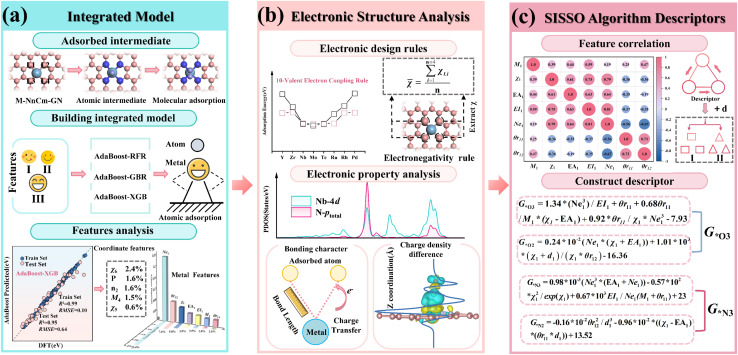
Overall workflow of this study. (a) An integrated model was established to extract the key features Ne_1_ and *χ*_6_. (b) The proposed electronegativity-regulated rule for interfacial valence-electron coupling, together with electronic-structure analysis, elucidates the microscopic mechanism of adsorption. (c) Adsorption-energy descriptor constructed by the SISSO algorithm.

This study employs a DFT-EL approach (Fig. 1), which illustrates the multi-stage design strategy for M–N–C-based SACs. Computational particulars are provided in SI Note 1; we therefore proceed directly to the results, beginning with structural stability.

## 3 Results and discussion

### 3.1 M–C–N structural construction

**Fig. 2:**
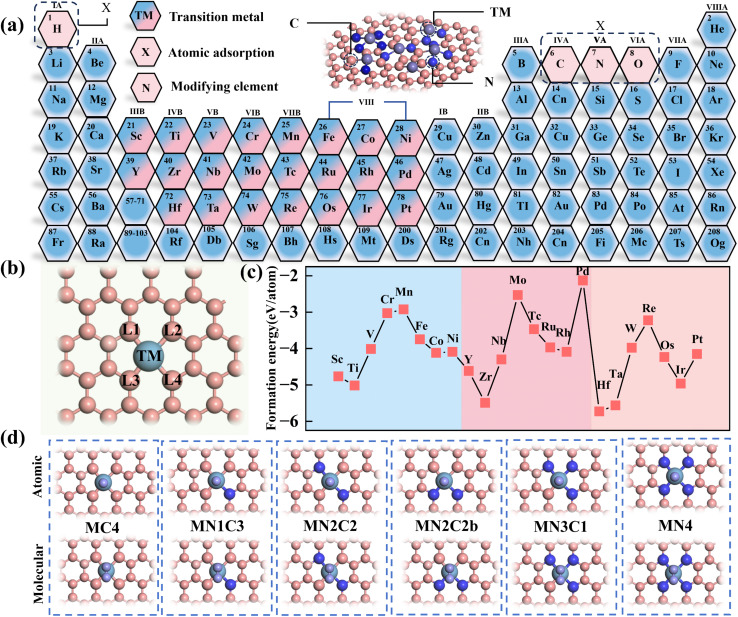
Schematic diagram of the M–C–N structure. (a) Types of metal dopant elements and adsorbed atoms. (b) The four N atom modification sites of MN4 (L1/L2/L3/L4). (c) Formation energies for N4 structures doped with 3d/4d/5d transition metals. (d) Structural diagrams of adsorbed atoms and molecules in different coordination environments.

In this study, six types of M–C–N (M = 3d, 4d and 5d (3d = Sc, Ti, V, Cr, Mn, Fe, Co and Ni), (4d = Y, Zr, Nb, Mo, Tc, Ru, Rh and Pd) and (5d = Hf, Ta, W, Re, Os, Ir and Pt)) structures, namely MC4, MN1C3, MN2C2, MN2C2b, MN3C1 and MN4 were constructed by substituting the four N atoms at the MN4 modification sites (L1/L2/L3/L4) (Fig. 2(a and b)).

The formation energy of the MN4 structure was calculated using the DFT method, and the structural stability was evaluated based on the formation energy. The results show that the formation energy of MN4 ranges from −2.15 eV to −5.73 eV, indicating strong stability. In MN4, the formation energy variations of the 3d, 4d and 5d doped systems all exhibit a significant W-shaped pattern, with the most stable structure occurring in the group IVB (Fig. 2(c)). The formation energy variations of the other five structures are shown in Fig. S1. Based on the structural stability mentioned above, the adsorption energies of different doped metals toward various adsorption intermediates (O/N/C/H) were calculated, and the effects of coordination environment variations on adsorption energy were analyzed.

Due to the differences in doped transition metals (TMs) and coordination environments, a wide variety of M–N–C structures (up to 138 types) were obtained. In particular, as the reaction process increases from the two-electron (HER) to the four-electron (ORR/OER), six-electron (NRR) and twelve-electron (CRR) reactions, the number of transition states for adsorbed intermediates increases significantly, and a complete exploration of the stability and catalytic performance of various M–N–C structures requires substantial computational resources. Therefore, a combined approach of DFT calculations and the EL method was employed, using the adsorption energies of atomic intermediates in various reactions as target values.

### 3.2 Ensemble learning

#### 3.2.1 Feature engineering

According to the variations in the M–N–C structures, three categories of features were constructed. Category I includes the intrinsic features of M, adsorbates (O/N/C/H), and coordinated N atoms, such as the atomic number (*n*), atomic mass (*M*), electronegativity (*χ*), electron affinity (*E*_A_), first ionization energy (*E*_I_), covalent radius (*θr*_11_) and calculated radius (*θr*_12_). Category II includes coordination environment features, including four N-doping sites (Z2, Z3, Z4 and Z5, where 0/1 represent doped/undoped, respectively), and the sum of the electronegativities of the four coordination atoms (C/N), denoted as *χ*_6_. Category III includes DFT-calculated features, specifically the formation energies (*P*) of six N-doped defective graphene structures. A dataset consisting of 210 adsorption energies of atomic intermediates (O, N, C and H) on M–N–C sites was designed based on the target values and used as the initial training samples for the model. All features were normalized to improve the efficiency of model training.

To improve the training accuracy of the ensemble model, feature selection was performed based on feature importance and Pearson correlation coefficients. The base models GBR, XGB and RFR of the ensemble model were used for pre-training. An ensemble model was developed using three base learners—GBR (Gradient Boosting Regressor), XGB (Extreme Gradient Boosting) and RFR (Random Forest Regressor)—for pre-training. The dataset was split into training (80%) and test (20%) partitions, and model predictive accuracy was assessed using the coefficient of determination (*R*^2^) and root-mean-square error (RMSE).

Fig. S3 shows the training results and the feature importance rankings of the three models. The results show that the *R*^2^ values of the training sets for GBR and XGB exceed 0.95, while the *R*^2^ values of the test sets are above 0.90. In contrast, the *R*^2^ values for the RFR model are 0.89 for the training set and 0.88 for the test set, indicating that the model performs relatively poorly on this dataset. This difference may stem from the fact that the RFR model, as a random forest, selects subsets of features and samples to build each tree, making the learning of each feature relatively independent and thus failing to fully capture the intrinsic patterns of the data.^54^ This also reflects the complexity of the dataset. Among them, Ne_1_ ranks the highest in importance across the three algorithms, while *χ*_6_ ranks the highest in the type II coordination environment features.

The Pearson correlation coefficients of 54 features were calculated (Fig. S4). The results show that the intrinsic features of nonmetals and metals exhibit weak correlations, while relatively strong correlations are observed with the coordination sites Z2–Z5. Features with high correlations |0.90| and relatively low importance rankings for the target were removed. Fig. S5 shows the final adsorption energy dataset retaining 15 features, including category I metal features: *M*_1_, *χ*_1_, *E*_A1_, *E*_I1_, Ne_1_, *θr*_11_ and *θr*_12_; category II adsorbed intermediate features: *E*_A0_, *E*_I0_ and *χ*_0_; and category III coordination environment features: *M*_4_, *n*_2_, *χ*_5_, *χ*_6_ and *P*.

#### 3.2.2 Construction of the integrated model

**Fig. 3:**
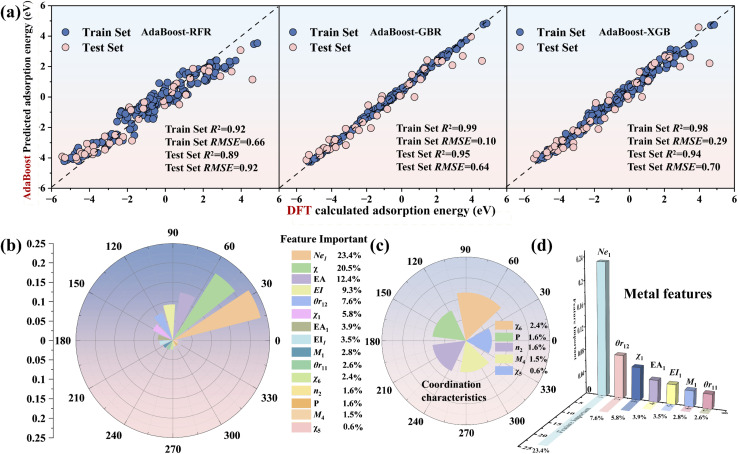
(a) The specific performance of the training and test sets for AdaBoost-RFR, AdaBoost-XGB and AdaBoost-GBR. (b) Feature importance ranking for AdaBoost-XGB. (c) The importance proportion of coordination environment features. (d) Feature importance ranking of the doped metal.

The optimized base learners from Section 3.2 were incorporated into the AdaBoost framework to construct the ensemble models (AdaBoost-RFR, AdaBoost-GBR and AdaBoost-XGB). For each ensemble model, fourfold cross-validation was employed to evaluate model performance. Fig. 3(a) shows the fitting performance of the three ensemble models for the intermediate adsorption energies (O/N/C/H) on M–N–C structures. The results show that the AdaBoost-XGB and AdaBoost-GBR models achieve an *R*^2^ of over 0.98 on the training set, with *R*^2^ values of 0.95 and 0.94 on the test set, respectively. The training set RMSE is 0.10 and 0.29, while the test set RMSE is 0.64 and 0.70, respectively. For the AdaBoost-RFR model, the *R*^2^ and RMSE values for the training set are 0.92 and 0.66, while those for the test set are 0.89 and 0.91. Compared with the other two ensemble models, its performance is relatively poorer, consistent with the pre-training results in Section 3.2; however, its accuracy has been significantly improved compared to the base models. By comprehensively comparing the *R*^2^ and RMSE of the three models, we select the optimal AdaBoost-XGB model for further feature analysis.

Fig. 3(c) shows the feature importance rankings for the AdaBoost-XGB model, where the metal valence electron Ne_1_ has the highest proportion (23.4%) and the electronegativity *χ*_0_ of the adsorbed intermediate ranks second (20.5%), indicating that the metal valence electron Ne_1_ is a key factor influencing the variation in adsorption energy. The average coordination electronegativity *χ*_6_ ranks the highest among the type II coordination environment features (2.4%), while the formation energy *P* of the N-doped defective graphene structure ranks second (1.6%), making them important features identified by the ensemble model for environmental variables.

To clarify the positive and negative effects of each feature on the adsorption energy, SHAP analysis was performed on the data (Fig. S6). Among them, the metal valence electron Ne_1_ and the electron affinity *E*_A1_ show a positive correlation with the adsorption energy, further confirming that valence electrons are key features for predicting adsorption energy. For the adsorbed intermediates, a larger electronegativity *χ*_0_ and electron affinity *E*_A0_ are favorable for adsorption, while a larger first ionization energy *E*_I0_ is unfavorable for adsorption. The defect feature *P* and the total electronegativity *χ*_6_ in the coordination environment show a positive influence on the adsorption energy.

The AdaBoost-GBR model was applied to estimate the adsorption energies of N and C intermediates for the remaining 342 M–N–C configurations. 40 MN4 (*M* = 3d, 4d and 5d) structures adsorbing O and N intermediates were selected for DFT calculations to verify the accuracy of the model (Fig. S7). The results indicate that the model achieves excellent predictive accuracy (*R*^2^ = 0.95) and demonstrates strong generalization performance. The analysis of the three base learners and AdaBoost-XGB indicates that Ne_1_ is the main factor influencing the adsorption energy of transition metal intermediates and the positive correlation between *χ*_6_ and adsorption energy indicates that the substrate environment is also one of the factors affecting the variation in adsorption energy. Therefore, we analyzed the variations in adsorption energy of M–N–C structures with respect to their valence electron numbers and coordination environments when adsorbing different reaction intermediates.

### 3.3 Electronegativity-controlled valence electron coupling rule

#### 3.3.1 Interfacial 10-electron coupling rule

**Fig. 4:**
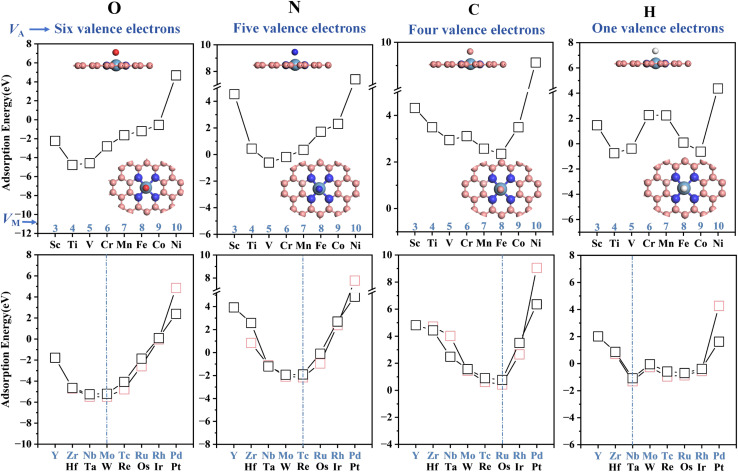
The adsorption energies and trends of different atomic (O/N/C/H) intermediates adsorbed on the MN4 surface. For 3d series metals, the most stable adsorption energies are typically found at the Ti and V positions. For the 4d and 5d doped metal series, the most stable adsorption configuration depends on the number of valence electrons of the adsorbed species.

We chose the widely studied MN4 (*M* = 3d, 4d and 5d) as a model and used DFT calculation data to analyze the adsorption energies of different adsorption atom intermediates (O/N/C/H) and their trends upon doping with different transition metals (Fig. 4 and Table S3). It was observed that upon 3d metal doping, the O/C/H adsorption energy trends exhibit a W-shaped profile, where the most stable configurations appear for Ti, V and Fe. The adsorption energy trend of N follows a V-shaped pattern, with the most stable configuration occurring at the metal V. For 4d and 5d metal doping, the adsorption energy variations of O, N and C exhibited a V-shaped trend. The 4d and 5d metal dopants exhibit comparable force distribution characteristics in the adsorption of intermediates. In the case of 3d metal doping, the adsorption energy of N exhibits a V-shaped fluctuation different from that of other adsorbates. This phenomenon originates from the pronounced spin polarization of 3d metal atoms and their strong coupling with the lone electron of the N atom.

The adsorption behavior of the adsorbates and doped metals is generated by the hybridization of partial, rather than all, valence electron orbitals, where the valence electron counts of O, N, C and H are 4, 3, 2 and 1, respectively.^49^ By observing the changes in the shared valence electrons of atomic intermediates and the most stable adsorption structures, it is found that as the partial valence electron number (*k*) decreases progressively O > N > C > H, the lowest point of the W-shaped curve shifts to the right (toward metals with higher *V*_M_). Specifically, a smaller adsorbed intermediate valence-electron count (*V*_A_) corresponds to a larger dopant-metal valence-electron count (*V*_M_), which resembles the stabilization mechanism whereby doped metals achieve equilibrium with adsorbed atomic intermediates through electronic compensation.

This further uncovers the pseudo-valence-electron compensation between the metal and the adsorbate, reflecting the correlation between the partial valence electron number (*k*) of the intermediate in the most stable adsorption state and the valence electron number (*V*_M_) of the active metal site. The results show that for the most stable adsorption structures of 4d and 5d transition metals, the total valence electrons of O, N and C shared with the doped metal sum to 10 (*V*_M_ + *k* = 10). Additionally, the total valence electrons for the adsorption of H sum to 6 (*V*_M_ + *k* = 6). This pattern is defined as the “10 valence electron coupling rule”. Due to the unpaired electrons in the outer d orbitals of 3d transition metals, they easily exhibit significant spin magnetic moments, which affect the electron filling of the metal atoms. If this spin polarization effect is suppressed, the adsorption states of 3d metals may follow this rule.

To validate the valence-electron matching rule, DFT calculations were performed to determine the adsorption energy of MN4 adsorbed on *P* (*k* = 3) and its variation trend (Fig. S7). The results indicate that the most stable adsorption structure is TcN4, consistent with the *V*_Tc_ + *k* = 10 rule satisfied by adsorbed nitrogen atoms, thereby demonstrating the accuracy of the valence electron rule for different reactions on MN4.

#### 3.3.2 Electronegativity regulation principle

**Fig. 5:**
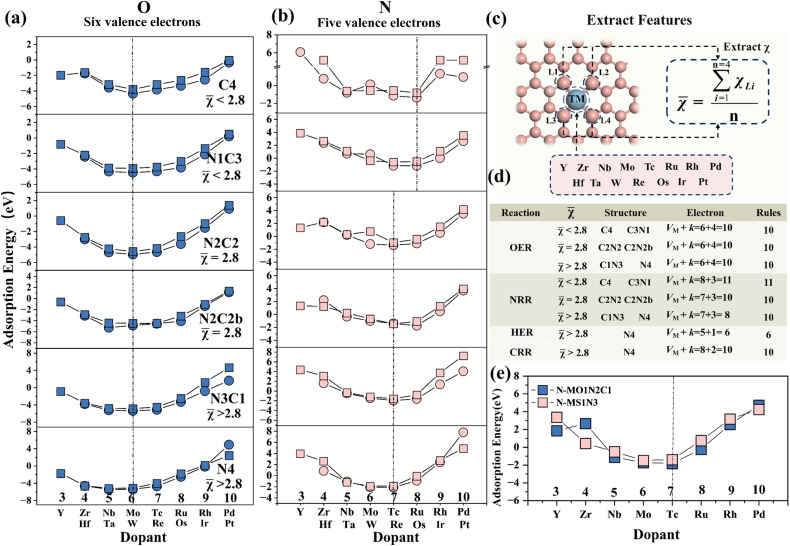
Rules for valence electron coupling in different coordination environments. (a and b) The variation of O and N adsorption energies on metals in six coordination environments (with different average electronegativities). (c) Method for calculating average electronegativity. (d) Valence-electron coupling rules with respect to average electronegativity for the NRR, OER, HER and CRR. (e) Randomly selected S and O modifications to validate the average electronegativity regulation mechanism of valence electrons.

To verify whether this rule can be extended, the adsorption energies for other M–N–C structure adsorption intermediates (O and N) were calculated (Table S4 and S5). The results indicate that the adsorption energies of O atoms on M–N–C (*M* = 4d and 5d) are shown in Fig. 5(a). Owing to the strong adsorption of the substrate toward the O intermediate, the adsorption energies of O on the transition-metal surfaces of MC4, MN1C3, MN2C2, MN2C2b, MN3C1 and MN4 all exhibited a V-shaped trend. Furthermore, they all satisfy the 10-valent electron coupling rule. The adsorption energy trend for the N intermediate adsorbed on MC4 and MN1C3 exhibits a W-shaped profile and does not satisfy the MN4 interfacial valence electron coupling rule. This is attributable to the influence of varying coordination environments on adsorption energy. According to the characteristic importance ranking in Section 3.3, among coordination environment characteristics, electronegativity *χ*_6_ emerges as a significant factor affecting adsorption energy. Consequently, the regulation of electronegativity on the most stable adsorption structure was analysed.

The average electronegativity of the coordination environment for each structure was calculated (
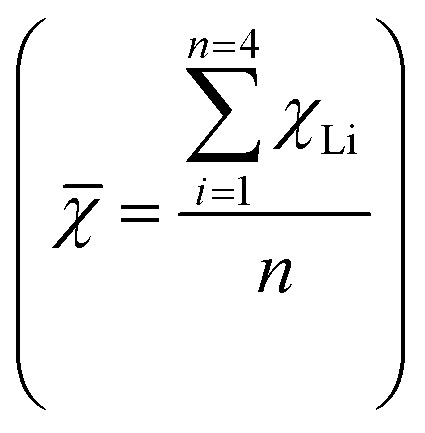
) to quantify the relationship between the average electronegativity and the variation in the calculated sum of *V*_M_ + *k*. The results in Fig. 5(b) show that as the number of N atoms in the coordination environment increases, the doped metal *V*_M_ in the most stable adsorption structure gradually decreases. For the N intermediates, the most stable adsorption sites are on Ru and Os (*V*_M_ + *k* = 11) when *χ̄* χ̄< 2.8 (MC4 and MN1C3). When *χ̄* = 2.8 (MN2C2 and MN2C2b) and *χ̄* χ̄> 2.8 (MN3C1 and MN4), the most stable adsorption sites are on Tc and Re (*V*_M_ + *k* = 10).

It can be inferred that by calculating the average electronegativity of the coordination environment, the valence electron rules for different reactions can be obtained, thereby predicting the doped metal for the most stable adsorption. As the electronegativity of the coordination environment increases, the doped metal in the most stable adsorption structure shifts to the left. This is attributed to the differences in the coordination environment and electronegativity of different configurations, which result in variations in the electron distribution at the metal center, thereby affecting the electron transfer and changes in adsorption energy during the adsorption process.

The adsorption energies of N on two randomly selected S- and O-modified M–N–C structures were calculated (Fig. 5(f)) to verify the aforementioned electronegativity-driven interface valence electron coupling rule. The results show that for *χ̄* χ̄> 2.8 (MS1N3 and MO1C1N2), the most stable adsorption site for the N intermediate is on Tc (*V*_Tc_ + *k* = 10). This is consistent with the pattern in Fig. 5(e), confirming the accuracy of the prediction based on the electronegativity-driven interface valence electron coupling rule.

In summary, the adsorption patterns of atomic intermediates for the OER and NRR have been revealed. However, the adsorption of molecular intermediates in the rate-determining step of the reaction is more critical. Therefore, further analysis is conducted on the intrinsic relationship between the valence electron number (*k*) of molecular intermediates interacting with metals and the doped metal (*V*_M_).

#### 3.3.3 RDS valence-electron coupling rule

**Fig. 6:**
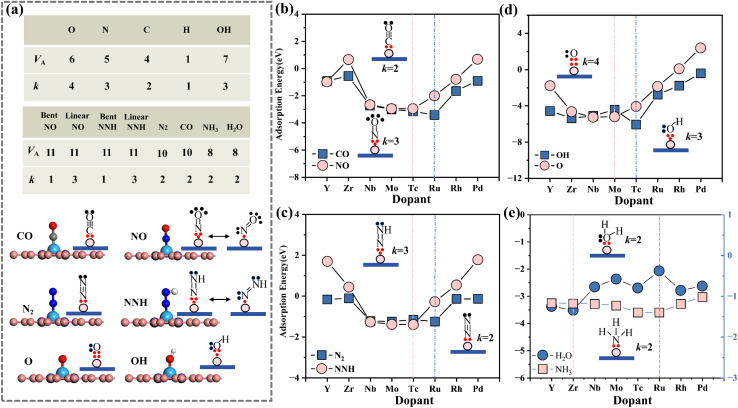
Verifying the adsorption patterns of molecules. (a) Schematic diagram of the partial electron count of the adsorbed intermediate and the interaction between MN4 and the adsorbed molecular intermediate. (b–e) The adsorption energy trends for NO, CO, N_2_, NNH, OH, H_2_O and NH_3_ adsorbed onto MN4.

Due to the influence of electronic orbital spin polarization on 3d metals, only 4d metal doping was considered when studying molecular intermediates. The adsorption energies of molecular intermediates on 4d-metal-doped MN4 systems were calculated (Table S7) to verify the consistency of the interface valence electron coupling rule with different reactive atoms and molecules. Fig. 6(a) shows the total number of valence electrons (*k*) required for the interaction between the adsorbed atoms and molecular intermediates with the metal. In the CRR process, CO couples with MN4 through the lone pair electrons on its carbon atom (Fig. 6(a)). In contrast, NO exhibits two possible interaction modes in the electrochemical environment: a single electron transfer in the bent configuration and a three-electron transfer in the linear configuration. This study only considers the interaction between linear NO and MN4 during the three-electron transfer. The results in Fig. 6(b) show that when *χ̄* χ̄>2.8 (MN4), the most stable adsorption sites for CO and NO are on Ru (*V*_Ru_ + *k* = 10) and Tc (*V*_Tc_ + *k* = 10), respectively. This is consistent with the results of the valence electron coupling rule for adsorbed atomic intermediates in Section 3.4, confirming the universality of the rule.

To verify the applicability of the 10-valence electron interface electron coupling rule to other molecules, the adsorption energies of H_2_O and NH_3_ on the MN4 structure were calculated (Table S6). The results show that NH_3_ follows the aforementioned interfacial 10-electron coupling rule and forms the most stable structure on Ru. Although H_2_O possesses an electron number (*k*) equivalent to that of CO in its interaction with the metal, its most stable configuration is not formed on Ru. This is attributed to the electrostatic interactions after H_2_O adsorption, which cause a redistribution of electron density, preventing H_2_O from satisfying the 10-valence electron interface electron coupling rule.

In the NRR/OER, the hydrogenation of N_2_ to form NNH and the conversion of OH to O (with a low enthalpy and considered the rate-determining step) occur.^55,56^ According to the 10-valence electron interface electron coupling rule (*V*_M_ + *k* = 10), it can be predicted that N_2_ is most stably adsorbed on Ru, while NNH and OH are most stably adsorbed on Tc. The results in Fig. 6(c and d) show that Ru adsorbs N_2_ to form the most stable adsorption structure, while the strongest adsorption of NNH and OH occurs at the Tc site. Taking the NRR in Fig. 6(e) as an example, when the number of valence electrons in the d orbital of transition metals ranges from 4 to 7, the smallest energy difference between N_2_ and NNH occurs at the Zr/Nb/Mo/Tc sites, with the smallest difference between Nb and Tc. This indicates that the energy required for the N_2_ → NNH transition is lower, facilitating the first reaction step. In the OER, a similar trend in adsorption energy variation is observed for the rate-determining step OH → O, as shown in Fig. 6(d). This further demonstrates that the 10-valent electron coupling rule at the interface also applies to the RDS (rate-determining step).

Through examples of M–N–C adsorption of atoms and molecules, we have preliminarily validated the basic concept for OER/NRR catalyst design and provided a reference for the screening of candidate materials. However, these rules have not yet fully revealed the relationship between the electronic properties of the doped metal (TM), the adsorbed intermediates (atomic or molecular), and the electronic structure of the coordination environment. Therefore, Section 3.4 systematically analyzes the electronic structures of the doped metals, adsorbed intermediates, and coordination environments to elucidate the intrinsic mechanism of the variation in catalyst adsorption energy.

### 3.4 Electronic property analysis

**Fig. 7:**
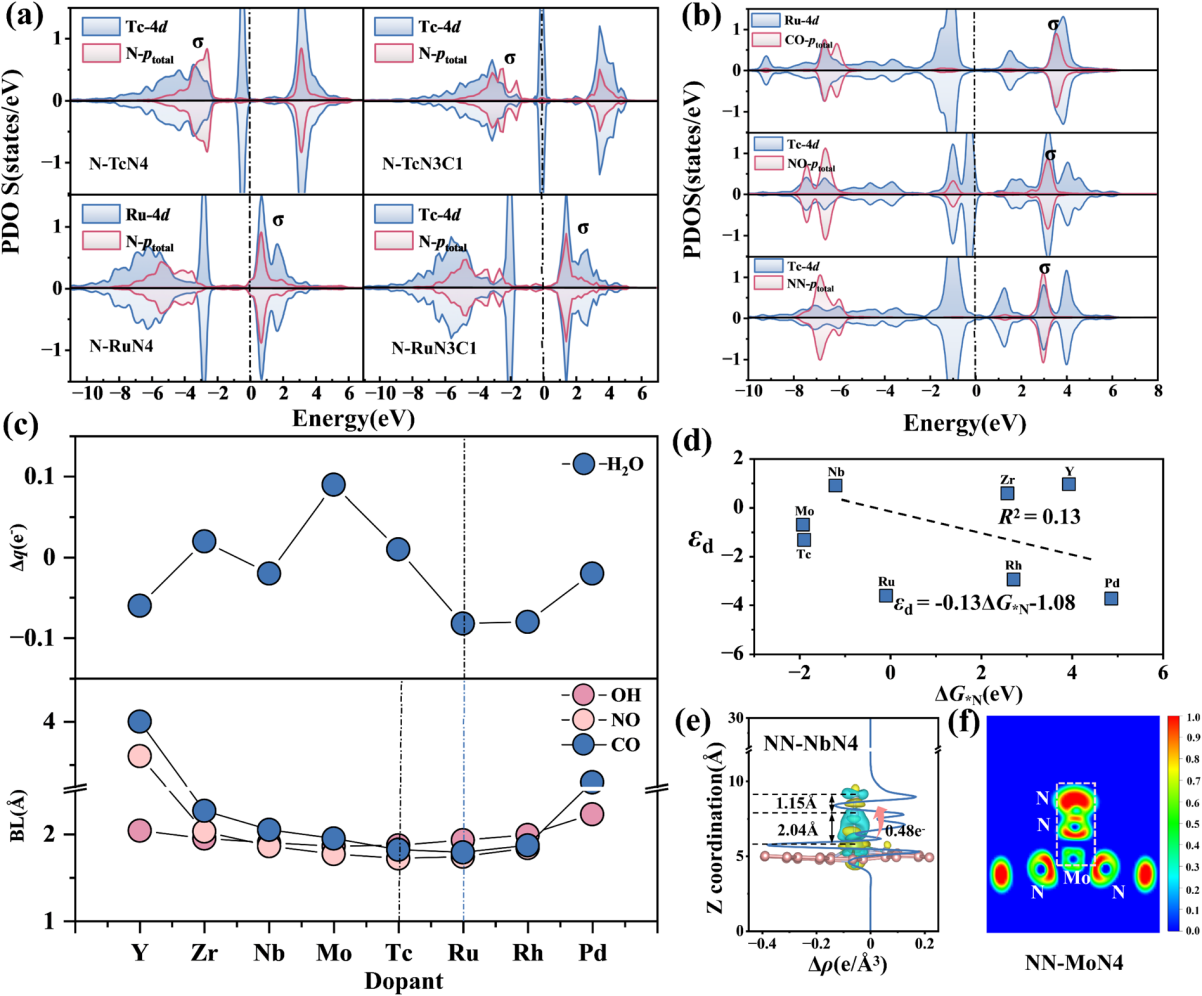
Electronic structure analysis. (a) The PDOS of MN3C1 and MN4 for N adsorption. (b) The PDOS of MN4 for NO, CO and NN adsorption. (c) Charge transfer behavior of the MN4 structure during H_2_O adsorption, and the variations in M–O, M–C and M–N bond distances when MN4 adsorbs OH, CO and NO. (d) The adsorption energy of N on MN4 and its corresponding fitting behavior within the d-band. (e) The three-dimensional charge density difference of NN-NbN4. (f) The ELF (Electron Localization Function) of MoN4 for NN adsorption.

To elucidate the intrinsic mechanism underlying the adsorption-strength variations governed by electronegativity-regulated interfacial valence-electron coupling, we analyzed the electronic structures associated with both atomic and molecular adsorption processes (atomic and molecular). Fig. 7(a) shows the projected density of states (PDOS) for N adsorption on MN3C1 and MN4 (M = Tc/Ru) structures. The results show that for the TcN3C1 and TcN4 substrates, strong interactions between N-p_total_ and Tc-4d exist in the ranges of −2.5 to −2.8 eV and −2.5 to −3.0 eV, respectively. The corresponding adsorption energies are −1.62 eV and −1.90 eV, both forming stable adsorption structures. Compared to Ru-4d (the most stable point of the coordination environment *χ̄* χ̄< 2.8) and Tc-4d (the most stable point of the coordination environment *χ̄* χ̄< 2.8), the orbital resonance peak of Nb-4d with N-p_total_ is higher and closer to the Fermi level, with stronger orbital hybridization, forming a stronger σ bond, thus resulting in the most stable adsorption (satisfying the law of 10 valence electron coupling).

Similar to MN3C1 and MN4 (*χ̄* > 2.8), for substrates with coordination environments of MC4 and MN1C3 (*χ̄* < 2.8) and MN2C2 and MN2C2b (*χ̄* = 2.8), the strongest orbital hybridization occurs between Ru-4d and N-p_total_ and between Tc-4d and N-p_total_ when adsorbing N intermediates (Fig. S9), consistent with the 11 and 10-valence electron coupling rules. For Tc doping, compared to environments *χ̄* < 2.8 and *χ̄* = 2.8, when the coordination environment is *χ̄* > 2.8, the energy level of Tc-d (−2.5 to −2.8 eV and −2.5 to −3.0 eV) is significantly higher, leading to greater electron transfer to the substrate and N, resulting in more stable adsorption. The influence of different coordination environments on the metal d-orbital electrons leads to differences in the adsorption strength of the same intermediate, resulting in different electronegativity control principles.

Further calculations were performed for the charge density difference and ELF local electron density in the OER (Zr-doped) (Fig. S15 and S16) and the NRR (Nb-doped) (Fig. S17 and S18). The results show that as the number of N atoms in the coordination environment increases and the average electronegativity increases, the total number of electrons transferred from the metal to the surroundings also increases. In addition, the charge density at the N site is significantly greater than that at the C site, indicating that the metal M transfers more electrons to the site with higher electronegativity. This further explains the reason for the shift of the valence electron rule with the average electronegativity.

The d-band center (*ε*_d_) of N-MN4 (M = 4d) was calculated, and unlike the traditional d-band center theory (which suggests strong adsorption near the Fermi level), the results in Fig. 7(d) show an *R*^2^ value of 0.15, indicating that the d-band center cannot be used to describe changes in adsorption energy. This is due to the change in electronegativity of the N-modified graphene substrate, which affects the charge distribution of the single atom, and the combined influence of strong π–π interactions, the restrictions of computational methods, and the constraints of experimental conditions, leading to a mismatch between *ε*_d_ and the variation in adsorption energy.

The PDOS of the adsorption intermediates (CO/NO/N2)-MN4 (M = 4d) for molecular adsorption was calculated (Fig. 7(b) and S12, S20). The results show that, compared to the other seven doped metals, Tc-4d exhibits strong orbital resonance with NO-ptotal on the left side of the Fermi level, forming a stable σ bond. NO-TcN4 forms the most stable adsorption structure 
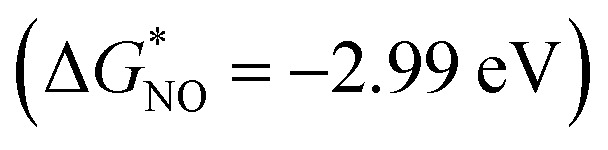
, which is consistent with the 10-valence electron coupling rule. When MN4 adsorbs CO, NO and OH intermediates, compared to the other structures, CO-RuN4, NO-TcN4 and OH-TcN4 form stable adsorption structures. The bond lengths between C-Ru, N-Tc and O-Tc are the shortest, indicating stronger interactions between them,^57^ which correspond to the 10 valence electron rules. Thus, the distance between the metal and the adsorbed molecular intermediate (*d*_1_) can be used as a descriptor for adsorption energy. Fig. 7(d) shows the charge transfer density difference of NN-NbN4. The results show that the metal Nb transfers the most electrons to NN (0.48e^−^), corresponding to the longest N–N bond (1.15 Å), which facilitates molecular activation, consistent with the results reported in the literature.^58^ The curve shows significant charge fluctuations near the interface, indicating that more electrons are transferred from the metal Nb in MN4 to the adsorbed intermediate N. Fig. 7(e) shows the electron localization function (ELF) between NN and Mo. The results indicate enhanced electron localization between NN and Mo, with a strong ionic bond formed between N and Mo.

To reveal the NRR activity of the MN4 structure, the PDOS of NNH was calculated (Fig. S21). The results show strong hybridization between NNH and Nb and Mo and Ru at the Fermi level, exhibiting a similar hybridization phenomenon to that observed with N_2_. The corresponding adsorption energies are as follows: NbN4: G_*NN_ = −1.22, G_*NNH_ = −1.27; MoN4: G_*NN_ = −1.26, G_*NNH_ = −1.39; TcN4: G_*NN_ = −1.16, G_*NNH_ = −1.40. The adsorption energies of the three are quite close, demonstrating good NRR catalytic activity. Since H_2_O forms the most stable structure with ZrN4, it does not follow the 10-valence electron coupling rule. Studies have shown that classical repulsion weakens the adsorption of H_2_O, while this electrostatic interaction can be offset by greater electron transfer.^23,57^ The variation of adsorption energy for NH_3_ on MN4 follows the 10-electron coupling rule, confirming that electron transfer can offset electrostatic repulsion. However, when M = Ru, RuN4 transfers the maximum number of electrons to H_2_O (−0.082e^−^), consistent with the 10-electron coupling rule. Thus, the factors affecting the adsorption intermediates should consider the synergistic effects of electron transfer and geometric structural characteristics to guide the design of catalysts.

In the case of atomic and molecular intermediate adsorption on M–N–C single metal catalysts, for the adsorption of atomic and molecular intermediates on M–N–C single-atom catalysts, the valence-electron coupling between the doped metal and the adsorbed intermediates can be analyzed by evaluating the average electronegativity of the coordination environment. Through the above study, we have derived a simplified descriptor (electronegativity-controlled valence electron coupling rule) that can be used for catalyst design. However, the above adsorption rules are only associated with the valence electron number of the doped metal and the electronegativity of its coordination environment and still fail to clearly reveal the adsorption energy relationship among the transition metal, the adsorbate, and the surrounding coordination structure. Therefore, there is an urgent need for in-depth research into the patterns of adsorption energy changes to provide more accurate guidance for catalyst screening.

### 3.5 Construction of adsorption energy descriptors

**Fig. 8:**
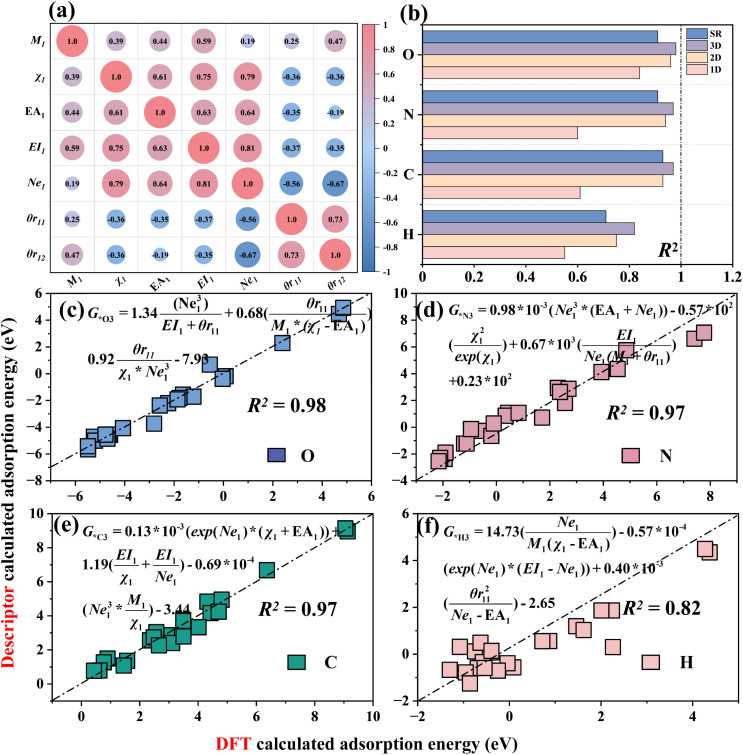
Pearson correlation coefficient and SISSO algorithm fitting *R*^2^. (a) Feature relationships after feature transformation. (b) The *R*^2^ predictions for various adsorption intermediates using the SISSO and SR algorithms (1D/2D/3D). (c–f) Comparison between the predicted results of 3D MN4-based catalyst descriptors and the outcomes obtained from DFT calculations.

By using the SISSO (Sure Independence Screening and Sparsity Operator) algorithm^59^ and SR (Symbolic Regression) algorithm,^60^ the adsorption energy data of (O, N, C and H)–M–N–C structures were fitted, respectively. Seven features, including *M*_1_, *χ*_1_, *E*_A1_, *E*_I1_, Ne_1_, *θr*_11_ and *θr*_12_, which have high feature importance as discussed in Section 3.2 (Fig. 8(a)), are selected as inputs for machine learning, with adsorption energy as the output.

Using the SISSO algorithm, 1D, 2D and 3D adsorption energy descriptors for MN4 adsorbing four intermediates (O/N/C/H) were constructed and compared with the SR algorithm (Fig. 8(b)), to select a descriptor that is both simple and accurate for predicting adsorption energy. The results show that the 3D descriptors of the SISSO algorithm for the adsorption energy of the four intermediates have the highest *R*^2^ values, which are 0.98 (O), 0.97 (N), 0.97 (C) and 0.82(H), indicating the highest accuracy. The SR descriptors also performed well for the adsorption of O, N, C and H, but the overall *R*^2^ of SR is lower than that of the 3D descriptors of SISSO. Moreover, the complexity of the four SR descriptors (Fig. S28) is higher than that of the 3D SISSO descriptors (Fig. 8(c–f)). Therefore, SISSO was selected as the adsorption energy descriptor for constructing the remaining M–N–C structure.

Fig. S23–S26 show the 1D, 2D and 3D SISSO adsorption energy descriptors for the adsorption of O/N/C/H on six M–N–C structures. The results show that as the dimensionality of the descriptors increases, the *R*^2^ value also increases, indicating that higher-dimensional adsorption energy descriptors have stronger predictive accuracy. The adsorption energy descriptors for O, N and C adsorption involve the valence electrons (Ne_1_^3^) of the doped metals, indicating that the valence electron number (Ne_1_) of the doped metal is the primary feature influencing the variation in adsorption energy. This corresponds to the key descriptor Ne_1_ identified in Section 3.2, further validating the reliability of the valence electron matching rule.

Moreover, surprisingly, after adding the bond length (*d*_1_) between the metal and the adsorption intermediate to the feature set, the *R*^2^ values of the SISSO descriptors for the adsorption energies of O and N (Fig. S27) significantly improved in 1D/2D/3D. The 1D descriptor *R*^2^ for MN4 adsorption of the O intermediate reached 0.86 (G_*O1_ = 0.77 × 10^−2^(exp(Ne_1_)/Ne_1_ × *d*_1_) − 4.25), an improvement of 0.02. The *R*^2^ value for the one-dimensional descriptor of MN4 adsorption of nitrogen reached 0.86 (G_*N1_ = −0.315 × 10^−2^ × *θr*_12_^2^/*d*_1_^3^ + 10.55), representing an improvement of 0.16. The results show that the distance from the doped metal to the adsorption intermediate can be an important feature to improve the predictive accuracy of the descriptors, which corresponds to the finding in Section 3.4 that bond length affects adsorption energy. These adsorption energy descriptors contribute to catalyst design, laying the foundation for the development of catalysts.

## 4 Conclusions

This study investigates single-atom catalysts supported on M-NnCm-GN and reveals the energy evolution patterns of intermediates in the nitrogen reduction reaction (NRR) and the oxygen evolution reaction (OER). By integrating the ensemble model AdaBoost-XGB (*R*^2^ = 0.95), the intrinsic mechanism of the interfacial valence-electron coupling rule regulated by the electronegativity of the coordination environment was systematically revealed. The DFT results indicate that during 3d transition-metal doping, the adsorbed intermediates tend to form more stable adsorption configurations with early transition metals such as Ti and V. For 4d and 5d metal doping, when electrons donated by the adsorbed intermediates (O, N, C and H) fill the d orbitals of the dopant metal, the adsorption configuration becomes most stable, thereby reflecting the 10-electron coupling rule. However, when the coordination environment changes, the original rule no longer applies. Therefore, we proposed the electronegativity-regulated interfacial valence electron coupling rule and elucidated the mechanism by which the coordination environment regulates the valence electron rule. The results show that when the average electronegativity is less than 2.8 (MC4 and MN1C3), equal to 2.8 (MN2C2 and MN2C2b) and greater than 2.8 (MN3C1 and MN4), they correspond to the 11-electron, 10-electron, and 10-electron coupling rules, respectively. DFT calculations show that the S and O modified M–N–C structures also follow the 10-valence electron coupling rule, further confirming the universality of this rule.

Furthermore, two catalysts for the NRR and OER were constructed to verify whether the molecular intermediates follow a similar rule. The results show that MN4 adsorption of NO/CO/OH/N_2_/NNH/NH_3_ intermediates satisfies the 10-electron coupling rule, which can quickly estimate the energy barrier of the rate-determining step (RDS), thereby facilitating the design of catalysts. Electronic structure analysis indicates that the dopant metal transfers electrons to atoms with greater electronegativity in the coordination environment. Subsequently, a simple descriptor for multidimensional adsorption intermediates was constructed using the SISSO algorithm. For the NRR, compared to the 1-dimensional descriptor (*R*^2^ = 0.60), the 3-dimensional descriptor (*R*^2^ = 0.97) has higher predictive accuracy and can be used for precise catalyst design. In addition, the introduction of the distance feature (*d*_1_) between the doped metal and the adsorbed intermediate improves the predictive accuracy of the model by approximately 17%. This method offers valuable perspectives for advancing machine learning methodologies in catalyst studies. This approach provides a novel research pathway for efficiently screening novel catalysts and significantly enhances the possibilities for future catalyst design.

## Author contributions

Hang Zhang: conceptualization, methodology, investigation, writing – original draft, visualization. Zishan Luo: data curation, methodology. Xi Sun: data curation, methodology. Yuhang Zhou: data curation. Wenhao Yan: formal analysis. Jiawei Li: visualization. Tao Zhang: visualization. Hong Cui: methodology, writing – review & editing. Weizhi Tian: writing – review & editing. Rong Feng: methodology. Hongkuan Yuan: investigation.

## Conflicts of interest

The authors declare no conflict of interest.

## Supplementary Material

SC-OLF-D6SC00422A-s001

## Data Availability

The data supporting this article have been included as part of the supplementary information (SI). Supplementary information: Table S1: formation energies (*E*_f_) of MN4 (M = 3d, 4d and 5d); Table S2: formation energies (*E*_f_) of M–N–C (M = 3d, 4d and 5d); Table S3: adsorption energies of O, N, C and H on M‑NnCm‑GN (M = 3d, 4d and 5d); Table S4: adsorption energy of N on M‑NnCm‑GN (M = 4d and 5d); Table S5: adsorption energy of O on M‑NnCm‑GN (M = 4d and 5d); Table S6: (M = 4d) energies and adsorption energies of adsorbed states; Table S7: adsorption energies of molecular intermediates (*NO, *CO, *NN *NNH, *OH, *OOH, *H_2_O, *NH_3_) on the MN4 structure (M = 4d). See DOI: https://doi.org/10.1039/d6sc00422a
