# Supplementary material for: Integrated learning-assisted design of metal–nitrogen–carbon single-atom catalysts: electronegativity regulates the coupling rules of interfacial valence electrons
Source: Chem Sci. 2026 May 5;17(23):11612–26. doi: 10.1039/d6sc00422a (PMC13142277; doi:10.1039/d6sc00422a)
Supplement: SC-017-D6SC00422A-s001 [file SC-017-D6SC00422A-s001.pdf]

Supplementary Information (SI) for Chemical Science.
This journal is © The Royal Society of Chemistry 2026

# Integrated learning-assisted design of metal-nitrogen-carbon single-atom catalysts: Electronegativity Regulates the Coupling Rules of Interfacial Valence Electrons

Supplementary Materials:

Supplementary Note 1

Supplementary Tables 1-10

Supplementary Figures 1-27

**Supplementary Note 1**

**DFT calculations**

The DFT calculations for SACs were performed using the Vienna Ab-initio Simulation Package (VASP)[1]. The electronic exchange-correlation interactions were treated using the Perdew-Burke-Ernzerhof (PBE) functional within the generalized gradient approximation (GGA) [2, 3]. The vacuum layer, force, and energy convergence for the M-N-C were set to 30 Å, 0.02 eV/Å, and $10^{-5}$ eV, respectively. All calculations considered spin polarization. For geometric optimization, the Brillouin zone was sampled using a Monkhorst-Pack k-point grid with a setting of 3×3×1. For electronic structure calculations, a k-point grid of 5×5×1 was used. The cutoff energy was set to 520 eV. A 5×5×1 supercell was used as the M-N-C substrate.

The formation energy of the M-N-C defect was calculated as follows:

$$E_{\mathrm{f}} = E_{\text{M-N-C}} + \mathrm{n} \times \mu_{\mathrm{C}} - (E_{Graphene} + \mu_{\mathrm{N}} + \mu_{\mathrm{M}})$$

where $E_{Graphene}$ and $E_{\text{M-N-C}}$ Energy of graphene structures and energy of N-modified graphene structures, respectively. $\mu_{\mathrm{C}}$, $\mu_{N}$, $\mu_{\mathrm{M}}$ ,correspond to the energies of the C atom, the coordinated environment N, the doped metal M, respectively.

The adsorption energy calculation of M–N–C structures was performed as follows.

$$\Delta E_{*\mathrm{X}} = E_{\mathrm{slab}*\mathrm{X}} - E_{\mathrm{slab}} - E_{\mathrm{X}}$$

Here, $E_{\mathrm{slab}*\mathrm{X}}$ , $E_{\mathrm{slab}}$ and $E_{\mathrm{X}}$ respectively represent the M-N-C adsorption intermediate X, the M-N-C substrate, and the intermediate energy.

**Base Learner Construction**

The machine learning models are implemented in a Python 3 environment using Scikit-Learn[4]. The Adsorption Energy model is built using Extreme Gradient Boosting (XGB)[5], Random Forest Regression (RFR) [6], Support Vector Regression (GBR) [7], and Symbolic Regression (SR) [8]. These algorithms are highly iterative and handle data with nonlinear relationships .By predicting feature–target relationships, SR helps analyze ML models. The results of the regression algorithms are evaluated using two statistical regression metrics: Root Mean Square Error (*RMSE*) and Coefficient of Determination ($R^2$), with calculation methods provided in Eq. S3-S4. The data is split into 80% training set and 20% testing set for model training.

**Detailed Procedure of the Ensemble Model:**

**Step1:** The strategy combining BIBD and MCC was used to systematically screen representative data points in the candidate doping systems, followed by density functional theory (DFT) calculations. Based on these calculation results and literature data, an integrated learning dataset targeting adsorption energy was constructed to ensure the comprehensiveness and diversity of the dataset.

**Step2:** Feature Engineering. By applying Pearson correlation coefficient and average feature importance analysis, highly correlated and redundant features were eliminated.

**Step3:** The AdaBoost ensemble model was used for prediction tasks, with Gradient Boosting Regression (XGB and GBR) and Random Forest Regression (RFR) selected as base learners integrated into the AdaBoost framework. To optimize model performance, hyperparameter tuning was performed for the three base learners. Grid

search combined with 4-fold cross-validation was used to explore the optimal combination of hyperparameters. The model performance was evaluated using two metrics: Root Mean Square Error (*RMSE*) and the coefficient of determination ($R^2$), with a focus on the impact of key hyperparameters on the training performance of the base learners. Finally, the optimal base learners and hyperparameter combination were selected to achieve the best prediction accuracy and stability of the model.

**Step4:** After optimizing the base learners, three AdaBoost ensemble models (AdaBoost-RFR, AdaBoost-GBR, AdaBoost-XGB) were constructed for adsorption energy, with a weight allocation strategy applied in each ensemble model to optimize its performance. In each training round, the sample weights are updated to focus on misclassified samples, and the weights of the base learners are adjusted according to their error rates. To further enhance model performance, grid search was used for fine-tuning the key hyperparameters in the AdaBoost framework. By systematically comparing the performance of different ensemble models in prediction tasks, the best model was selected to ensure prediction accuracy and stability.

**Step5:** To reveal the prediction mechanism of the ensemble model, an interpretability study was conducted using SHAP (SHapley Additive exPlanations) analysis and feature importance evaluation.

Ensemble Learning Model Evaluation Criteria: The coefficient of determination ($R^2$) and the RMSE are defined as follows:

$$R^2 = 1 - \frac{\sum_{i=1}^{n}(y_i - \overline{y})^2}{\sum_{i=1}^{n}(y_i' - \overline{y})^2}$$

$$RMSE = \sqrt{\frac{1}{n}\sum_{i=1}^{n}(y_i - \mathrm{y}_i')^2}$$

Where $y_i$ is the difference between the actual and corresponding predicted values, and $y_i'$ is the average of the true values. The Ensemble Learning model performs best when $R^2$ is close to 1 and *RMSE* is close to 0V.

**SISSO algorithm**

The operation process of the SISSO algorithm is mainly divided into two stages: feature screening and sparsification model construction[9].

Sure Independence Screening (SIS): First, a set of potential descriptors is generated from a large pool of features, which are usually generated by a series of mathematical operations (e.g., addition, multiplication, logarithm, etc.) from atomic properties, geometrical parameters, etc. The SIS step filters out the most relevant features from these features with respect to the target variables (e.g., material properties) through correlation analysis, which reduces the spatial dimensions of the features. Reducing the feature space dimension.

Sparsifying Operator (SO): Among the filtered features, SO further extracts a small number of the most representative descriptors, usually using the L0 paradigm to construct a sparse model. This step is optimized to ensure that the retained features have the maximum impact on the target variables while maintaining the simplicity of the model.

Ultimately, the five after feature engineering are used as the eigenvalues, and the adsorption energy is used as the target values as inputs into the SISSO model, outputting a linear model with a high degree of interpretability.

**Table S1 Formation Energies ($E_f$) of MN4(M= 3*d*, 4*d* and 5*d*).**

| 3*d* | $E_f$ | 4*d* | $E_f$ | 5*d* | $E_f$ |
|---|---|---|---|---|---|
| Sc | −4.76 | Y | −4.61 | Hf | −5.73 |
| Ti | −5.02 | Zr | −5.49 | Ta | −5.56 |
| V | −4.01 | Nb | −4.30 | W | −3.98 |
| Cr | −3.03 | Mo | −2.53 | Re | −3.22 |
| Mn | −2.92 | Tc | −3.47 | Os | −4.23 |
| Fe | −3.75 | Ru | −3.97 | Ir | −4.96 |
| Co | −4.12 | Rh | −4.09 | Pt | −4.15 |
| Ni | −4.09 | Pd | −2.13 | | |

**Table S2 Formation Energies ($E_f$) of M-N-C(M= 3*d*, 4*d* and 5*d*).**

| 4d | MC4 | MN1C3 | MN2C2 | MN2C2b | MN3C1 |
|---|---|---|---|---|---|
| Y | 2.71 | 0.73 | −0.87 | −0.84 | −2.55 |
| Zr | −0.25 | −1.63 | −2.97 | −2.83 | −3.85 |
| Nb | −0.22 | −1.27 | −2.06 | −1.96 | −2.82 |
| Mo | 1.16 | 0.19 | −0.32 | −0.34 | −0.98 |
| Tc | 0.12 | −0.56 | −1.06 | −0.64 | −1.59 |
| Ru | −0.22 | −0.71 | −1.59 | −1.02 | −2.01 |
| Rh | 0.90 | −0.08 | −0.83 | −0.66 | −3.50 |
| Pd | 3.68 | 2.35 | 1.10 | 0.94 | −3.08 |
| 5d | MC4 | MN1C3 | MN2C2 | MN2C2b | MN3C1 |
| Hf | −0.34 | −1.68 | −3.01 | −2.95 | −3.91 |
| Ta | −1.37 | −2.38 | −3.20 | −2.67 | −3.89 |
| W | −0.45 | −1.24 | −1.84 | −1.97 | −2.44 |
| Re | −0.09 | −0.61 | −1.07 | −1.14 | −1.46 |
| Os | −0.90 | −1.28 | −2.02 | −1.46 | −2.31 |
| Ir | −0.47 | −1.33 | −2.08 | −2.02 | −2.88 |
| Pt | 0.90 | −0.19 | −1.14 | −1.40 | −2.32 |

**Table S3 Adsorption energies of O, N, C and H on M-NnCm-GN(M= 3*d*, 4*d* and 5*d*).**

| Metal | O-MN4 | N-MN4 | C-MN4 | H-MN4 |
|---|---|---|---|---|
| Sc | −2.24 | 4.54 | 4.32 | 1.46 |
| Ti | −4.78 | 0.44 | 3.49 | −0.75 |
| V | −4.59 | −0.61 | 2.94 | −0.39 |
| Cr | −2.81 | −0.19 | 3.11 | 2.26 |
| Mn | −1.64 | 0.35 | 2.57 | 2.24 |
| Fe | −1.21 | 1.71 | 2.35 | 0.09 |
| Co | −0.55 | 2.3 | 3.49 | −0.63 |
| Ni | 4.68 | 7.41 | 9.13 | 4.37 |
| Y | −1.78 | 3.93 | 4.81 | 2.01 |
| Zr | −4.65 | 2.57 | 4.43 | 0.87 |
| Nb | −5.25 | −1.21 | 2.48 | −1.08 |
| Mo | −5.20 | −1.93 | 1.57 | −0.04 |
| Tc | −4.07 | −1.9 | 0.9 | −0.58 |
| Ru | −1.86 | −0.1 | 0.77 | −0.69 |
| Rh | 0.09 | 2.71 | 3.5 | −0.39 |
| Pd | 2.39 | 4.86 | 6.36 | 1.62 |
| Hf | −4.70 | 0.83 | 4.71 | 0.72 |
| Ta | −5.48 | −1.11 | 4.02 | −1.3 |
| W | −5.49 | −2.1 | 1.45 | −0.25 |
| Re | −4.78 | −2.16 | 0.62 | −0.95 |
| Os | −2.58 | −0.95 | 0.43 | −0.86 |
| Ir | −0.23 | 2.38 | 2.66 | −0.54 |
| Pt | 4.85 | 7.77 | 9.05 | 4.27 |

**Table S4** Adsorption energy of N on M-NnCm-GN(M= 4*d* and 5*d*).

| 4d | N-MC4 | N-MN1C3 | N-MN2C2 | N-MN2C2b | N-MC1N3 |
|---|---|---|---|---|---|
| Y | 6.08 | 3.86 | 1.31 | 1.31 | 4.33 |
| Zr | 0.76 | 2.64 | 2.19 | 1.23 | 3.07 |
| Nb | −0.81 | 1.09 | 0.28 | 0.20 | −0.35 |
| Mo | 0.13 | −0.4 | 0.74 | −0.69 | −1.24 |
| Tc | −1.14 | −0.6 | −0.98 | −1.47 | −1.62 |
| Ru | −1.36 | −0.53 | −0.45 | −1.10 | −0.82 |
| Rh | 1.32 | 1.08 | 1.47 | 1.26 | 3.71 |
| Pd | 0.94 | 3.52 | 4.15 | 3.95 | 7.22 |
| 5d | N-MC4 | N-MC3N1 | N-MC2N2 | N-MC2N2b | N-MC1N3 |
| Hf | 5.17 | 2.36 | 2.16 | 2.19 | 1.56 |
| Ta | −0.61 | 0.70 | 0.21 | −0.36 | −0.50 |
| W | −0.56 | 0.62 | −1.19 | −1.03 | −1.50 |
| Re | −0.56 | −1.16 | −1.40 | −1.42 | −2.00 |
| Os | −0.81 | −1.18 | −1.12 | −1.76 | −1.65 |
| Ir | 5.18 | 0.01 | 0.52 | 0.45 | 1.34 |
| Pt | 5.17 | 2.64 | 3.50 | 3.67 | 4.02 |

**Table S5** Adsorption energy of O on M-NnCm-GN(M= 4*d* and 5*d*).

| 4d | N-MC4 | N-MN1C3 | N-MN2C2 | N-MN2C2b | N-MN3N1 |
|---|---|---|---|---|---|
| Y | −1.98 | −0.82 | −0.60 | −0.63 | −0.90 |
| Zr | −1.63 | −2.23 | −2.77 | −2.93 | −3.62 |
| Nb | −3.18 | −3.88 | −4.25 | −4.45 | −4.85 |
| Mo | −3.79 | −3.92 | −4.60 | −4.49 | −4.91 |
| Tc | −3.18 | −3.76 | −4.19 | −4.54 | −4.55 |
| Ru | −2.63 | −3.02 | −2.64 | −3.19 | −2.55 |
| Rh | −1.60 | −1.36 | −0.99 | −1.08 | 1.19 |
| Pd | −0.06 | 0.48 | 1.38 | 1.33 | 4.60 |
| 5d | N-MC4 | N-MC3N1 | N-MC2N2 | N-MC2N2b | N-MC1N3 |
| Hf | −1.75 | −2.43 | −3.02 | −3.13 | −3.84 |
| Ta | −3.57 | −4.33 | −4.70 | −5.32 | −5.29 |
| W | −4.35 | −4.48 | −4.98 | −4.86 | −5.43 |
| Re | −3.83 | −4.31 | −4.66 | −4.58 | −5.17 |
| Os | −3.34 | −3.83 | −3.62 | −4.13 | −3.34 |
| Ir | −2.54 | −2.13 | −1.54 | −1.40 | −0.85 |
| Pt | −0.33 | 0.20 | 0.91 | 1.09 | 1.58 |

**Table S6** (M= 4d) energies and adsorption energies of adsorbed states.

| 4d | MO1C1N2($E_f$) | N-MO1C1N2($E_{*N}$) | MS1N3($E_f$) | N-MS1N3($E_{*N}$) |
|---|---|---|---|---|
| Y | −2.58 | 1.84 | −1.34 | 3.36 |
| Zr | −3.60 | 2.65 | −2.26 | 0.42 |
| Nb | −2.40 | −1.13 | −2.25 | −0.52 |
| Mo | −0.75 | −1.74 | 0.36 | −1.50 |
| Tc | −1.62 | −1.84 | −0.59 | −1.40 |
| Ru | −2.14 | −0.24 | −1.35 | 0.74 |
| Rh | −2.26 | 2.55 | −1.59 | 3.16 |
| Pd | −0.50 | 4.72 | 0.96 | 4.23 |

**Table S7** MN4 (M= 4*d*) energies and adsorption energies of adsorbed states.

| 4d | *NO | *CO | *NN | *NNH | *OH | *OOH | *$H_2O$ | *$NH_3$ |
|---|---|---|---|---|---|---|---|---|
| Y | −0.98 | −0.92 | −0.17 | 1.70 | −4.57 | −3.62 | −3.38 | −1.17 |
| Zr | 0.66 | −0.55 | −0.11 | 0.44 | −5.37 | −6.79 | −3.52 | −1.18 |
| Nb | −2.67 | −2.73 | −1.22 | −1.27 | −5.11 | −8.38 | −2.66 | −1.19 |
| Mo | −2.96 | −3.00 | −1.25 | −1.39 | −4.43 | −7.18 | −2.37 | −1.23 |
| Tc | −2.99 | −3.16 | −1.16 | −1.40 | −6.06 | −5.17 | −2.70 | −1.40 |
| Ru | −2.01 | −3.42 | −1.25 | −0.28 | −2.76 | −2.83 | −2.07 | −1.40 |
| Rh | −0.80 | −1.65 | −0.14 | 0.54 | −1.78 | −0.36 | −2.79 | −1.19 |
| Pd | 0.69 | −0.90 | −0.14 | 1.78 | −0.41 | 0.64 | −2.63 | −1.02 |

**Table S8** Features data. *M*: atomic mass, $\chi$: electronegativity, EA: electron affinity energy, *EI*: first ionisation energy, *Ne*: outermost electron, $\theta r_{11}$: Covalent radius, $\theta r_{12}$: Calculated radius.

| Atom | $M_1$ | $X_1$ | $EA_1$ | $EI_1$ | $Ne_1$ | $\theta r_{11}$ | $\theta r_{12}$ |
|---|---|---|---|---|---|---|---|
| Sc | 44.96 | 1.36 | 0.19 | 6.56 | 3 | 144 | 184 |
| Ti | 47.87 | 1.54 | 0.09 | 6.83 | 4 | 136 | 176 |
| V | 50.94 | 1.63 | 0.53 | 6.75 | 5 | 125 | 171 |
| Cr | 52.00 | 1.66 | 0.68 | 6.77 | 6 | 127 | 166 |
| Mn | 54.94 | 1.55 | 0.97 | 7.43 | 7 | 139 | 161 |
| Fe | 55.85 | 1.83 | 0.15 | 7.90 | 8 | 125 | 156 |
| Co | 58.93 | 1.88 | 0.66 | 7.88 | 9 | 126 | 152 |
| Ni | 58.69 | 1.91 | 1.16 | 7.64 | 10 | 121 | 149 |
| Y | 88.91 | 1.22 | 0.31 | 6.22 | 3 | 162 | 212 |
| Zr | 91.22 | 1.33 | 0.43 | 6.63 | 4 | 148 | 206 |
| Nb | 92.91 | 1.60 | 0.89 | 6.76 | 5 | 137 | 198 |
| Mo | 95.96 | 2.16 | 0.75 | 7.09 | 6 | 145 | 190 |
| Tc | 96.91 | 1.90 | 0.55 | 7.28 | 7 | 156 | 183 |
| Ru | 101.07 | 2.20 | 1.05 | 7.36 | 8 | 126 | 178 |
| Rh | 102.91 | 2.28 | 1.14 | 7.46 | 9 | 135 | 173 |

| | | | | | | | |
|---|---|---|---|---|---|---|---|
| Pd | 106.42 | 2.20 | 0.56 | 8.34 | 10 | 131 | 169 |
| Hf | 178.49 | 1.30 | 0.63 | 6.83 | 4 | 150 | 208 |
| Ta | 180.95 | 1.50 | 0.32 | 7.55 | 5 | 138 | 200 |
| W | 183.85 | 2.36 | 0.82 | 7.86 | 6 | 146 | 193 |
| Re | 186.21 | 1.90 | 0.38 | 7.83 | 7 | 159 | 188 |
| Os | 190.23 | 2.20 | 1.08 | 8.70 | 8 | 128 | 185 |
| Ir | 192.22 | 2.2 | 1.56 | 9.10 | 9 | 137 | 180 |
| Pt | 195.08 | 2.28 | 2.13 | 9.00 | 10 | 128 | 177 |

**Table S9** Model parameters of the SISSO algorithm.

| | Parameters |
|---|---|
| SISSO | ptype=1，ntask=1<br>task_weighting=1，scmt=.false.<br>desc_dim=1，nsample= 999<br>! nsample = (n1, n2, ...) ，restart=0<br>nsf= 4，ops='(+)(-)(*)(/)(^-1)(^2) (^3) '<br>fcomplexity=3，funit=(1:7)<br>fmax_min=1e-3，fmax_max=1e-5<br>nf_sis=400，method_so= 'L0'<br>nl1l0= 1，fit_intercept=.true.<br>metric= 'RMSE'，nmodels=100<br>! isconvex=(1,1,...)，bwidth=0.001 |

**Table S10 Optimal Hyperparameters Selected for the Four Machine Learning Models**.

| Model | AdaBoost-RFR | AdaBoost-XGB | AdaBoost-GBR | SR |
|---|---|---|---|---|
| Optimal Hyperparameter | 'learning_rate': 0.003, 'n_estimators':3000, cv=4 | 'learning_rate':0.01, 'n_estimators':50, cv=4 | 'learning_rate':0.1, 'n_estimators':50, cv=4 | loop="MultiMutateLoop" pop=600, gen=50, add_coef=0.7, hall=3, re_hall=5, max_value=8, random_state=i |

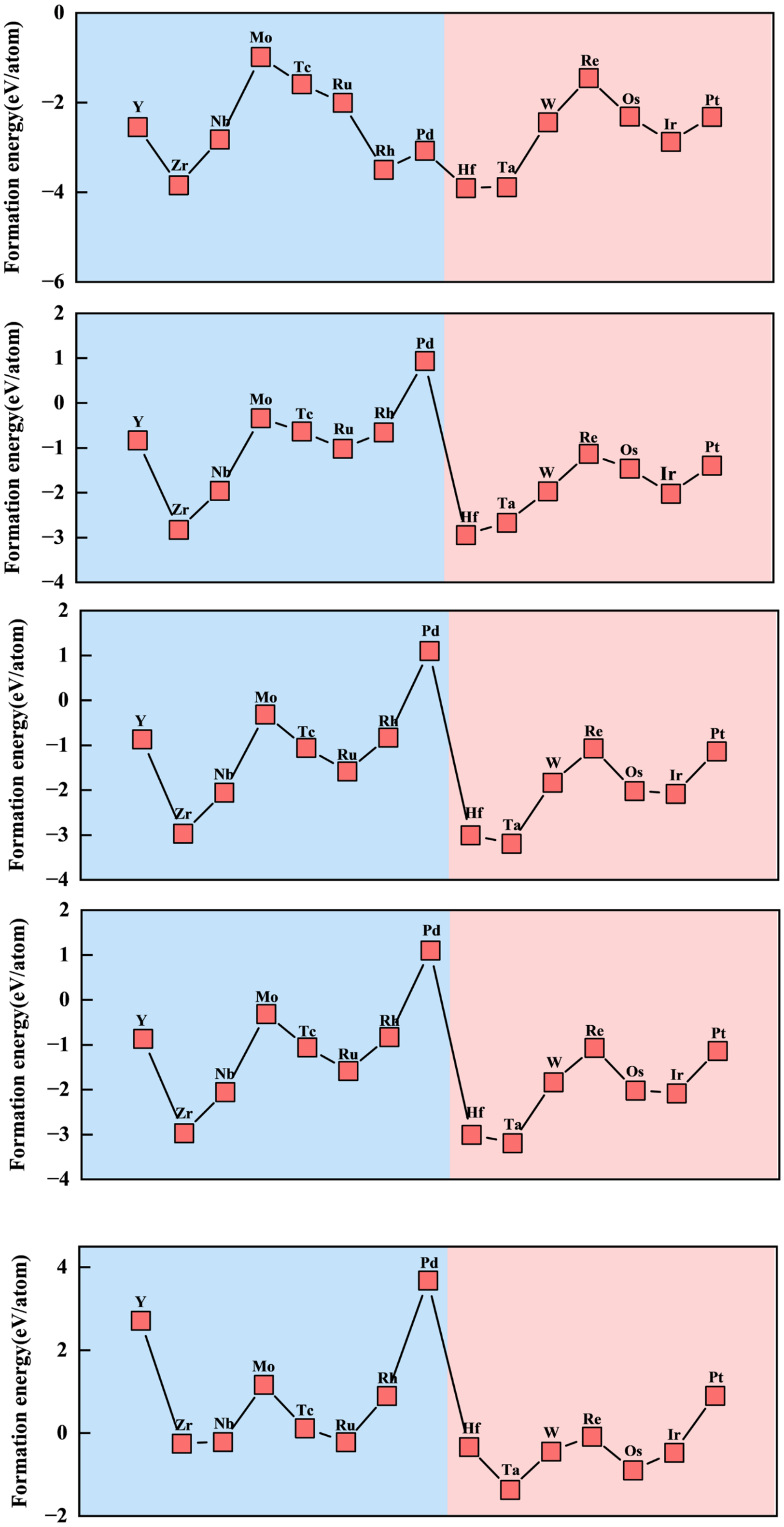


**Fig. S1** Formation energy of MC4, MN1C3, MN2C2, MN2C2b, MN3C1 and MN4**.**

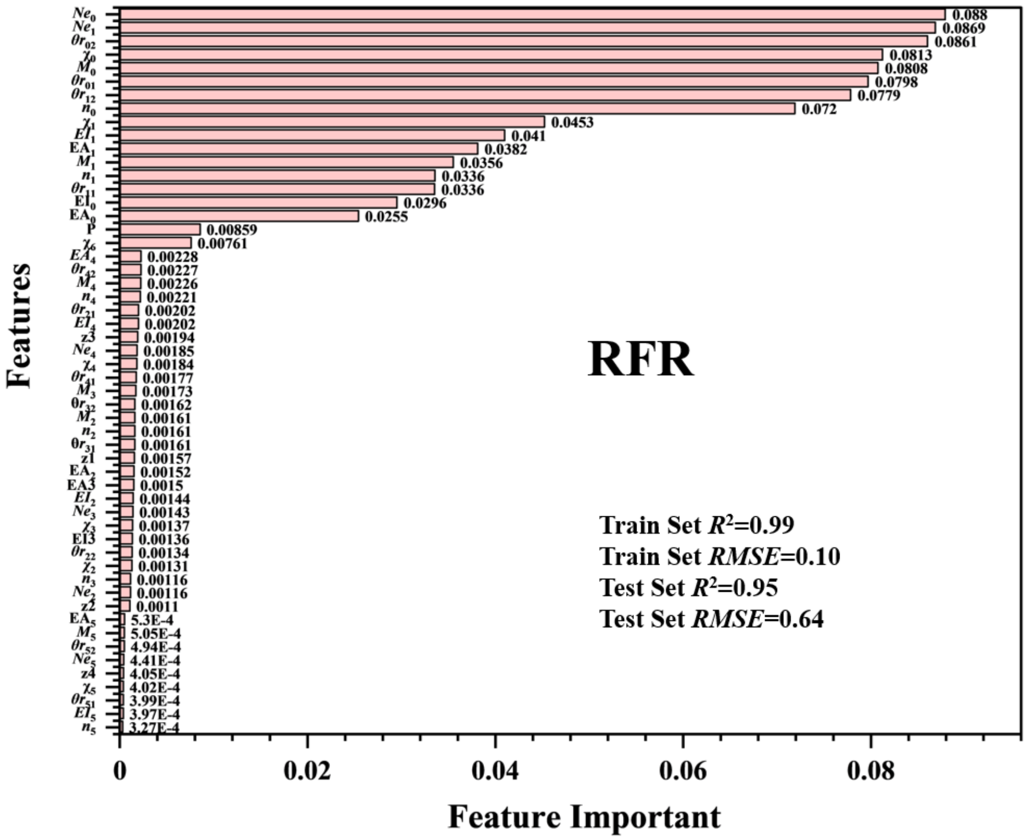

Features
RFR
Train Set $R^2$=0.99
Train Set *RMSE*=0.10
Test Set $R^2$=0.95
Test Set *RMSE*=0.64
0
0.02
0.04
0.06
0.08
Feature Important

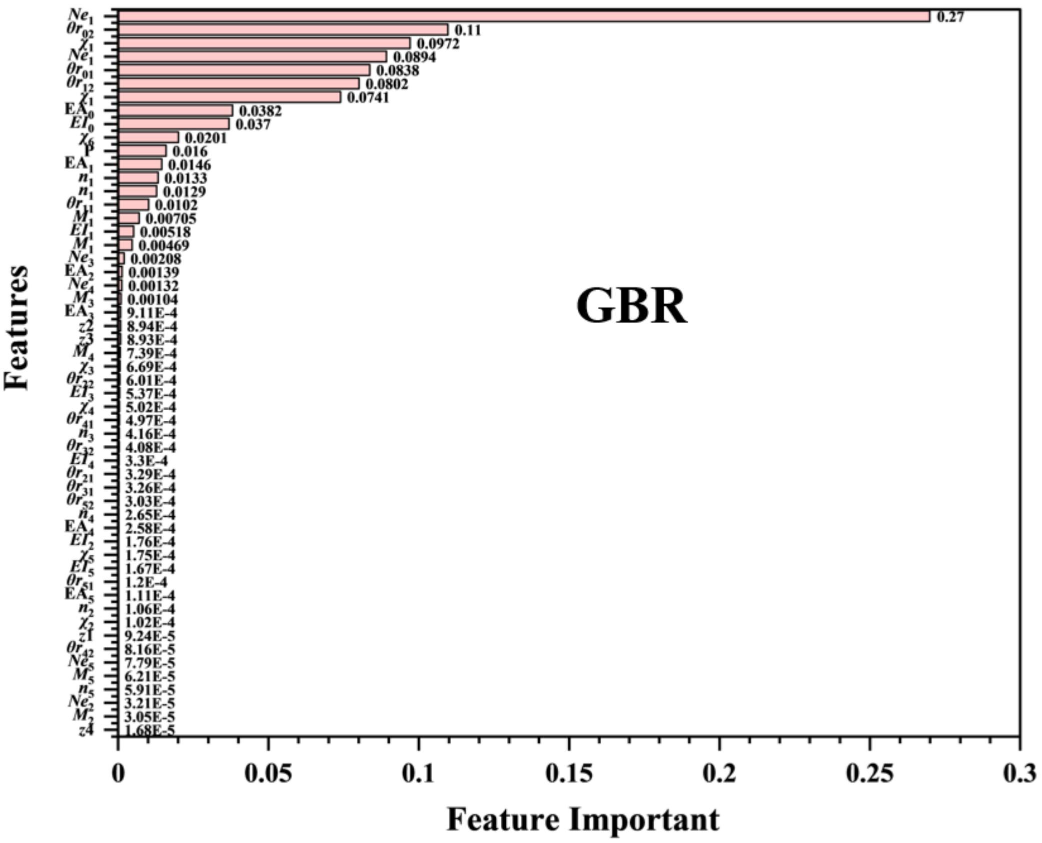
GBR
Features
Feature Important
0
0.05
0.1
0.15
0.2
0.25
0.3
$Ne_1$ 0.27
$\theta r_{02}$ 0.11
$\chi_1$ 0.0972
$Ne_1$ 0.0894
$\theta r_{01}$ 0.0838
$\theta r_{12}$ 0.0802
$\chi_1$ 0.0741
$EA_0$ 0.0382
$EI_0$ 0.037
$\chi_6$ 0.0201
P 0.016
$EA_1$ 0.0146
$n_1$ 0.0133
$n_1$ 0.0129
$\theta r_{11}$ 0.0102
$M_1$ 0.00705
$EI_1$ 0.00518
$M_1$ 0.00469
$Ne_3$ 0.00208
$EA_2$ 0.00139
$Ne_4$ 0.00132
$M_3$ 0.00104
$EA_3$ 9.11E-4
z2 8.94E-4
z3 8.93E-4
$M_4$ 7.39E-4
$\chi_3$ 6.69E-4
$\theta r_{22}$ 6.01E-4
$EI_3$ 5.37E-4
$\chi_4$ 5.02E-4
$\theta r_{41}$ 4.97E-4
$n_3$ 4.16E-4
$\theta r_{32}$ 4.08E-4
$EI_4$ 3.3E-4
$\theta r_{21}$ 3.29E-4
$\theta r_{31}$ 3.26E-4
$\theta r_{52}$ 3.03E-4
$n_4$ 2.65E-4
$EA_4$ 2.58E-4
$EI_2$ 1.76E-4
$\chi_5$ 1.75E-4
$EI_5$ 1.67E-4
$\theta r_{51}$ 1.2E-4
$EA_5$ 1.11E-4
$n_2$ 1.06E-4
$\chi_2$ 1.02E-4
z1 9.24E-5
$\theta r_{42}$ 8.16E-5
$Ne_5$ 7.79E-5
$M_5$ 6.21E-5
$n_5$ 5.91E-5
$Ne_2$ 3.21E-5
$M_2$ 3.05E-5
z4 1.68E-5

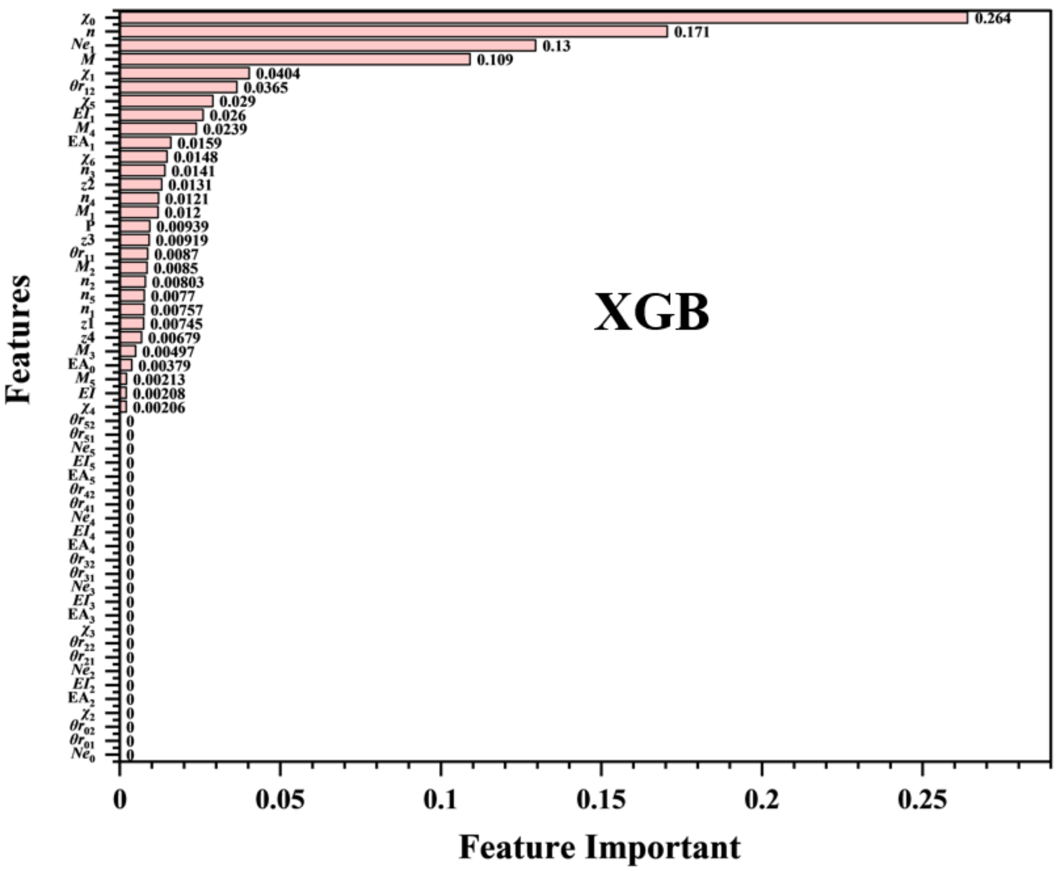


**Fig. S2** Formation energy of MC4, MN1C3, MN2C2, MN2C2b, MN3C1 and MN4**.**

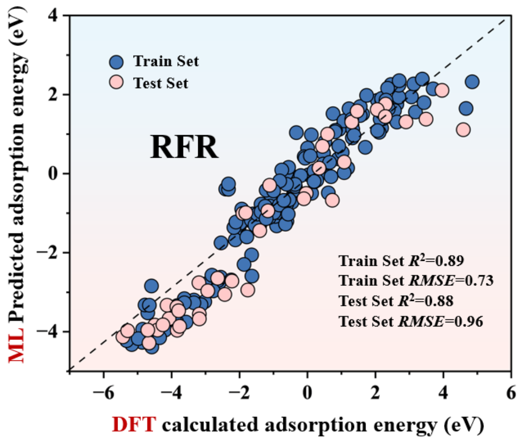


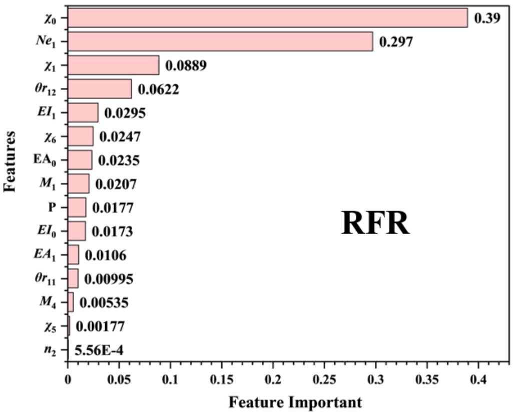

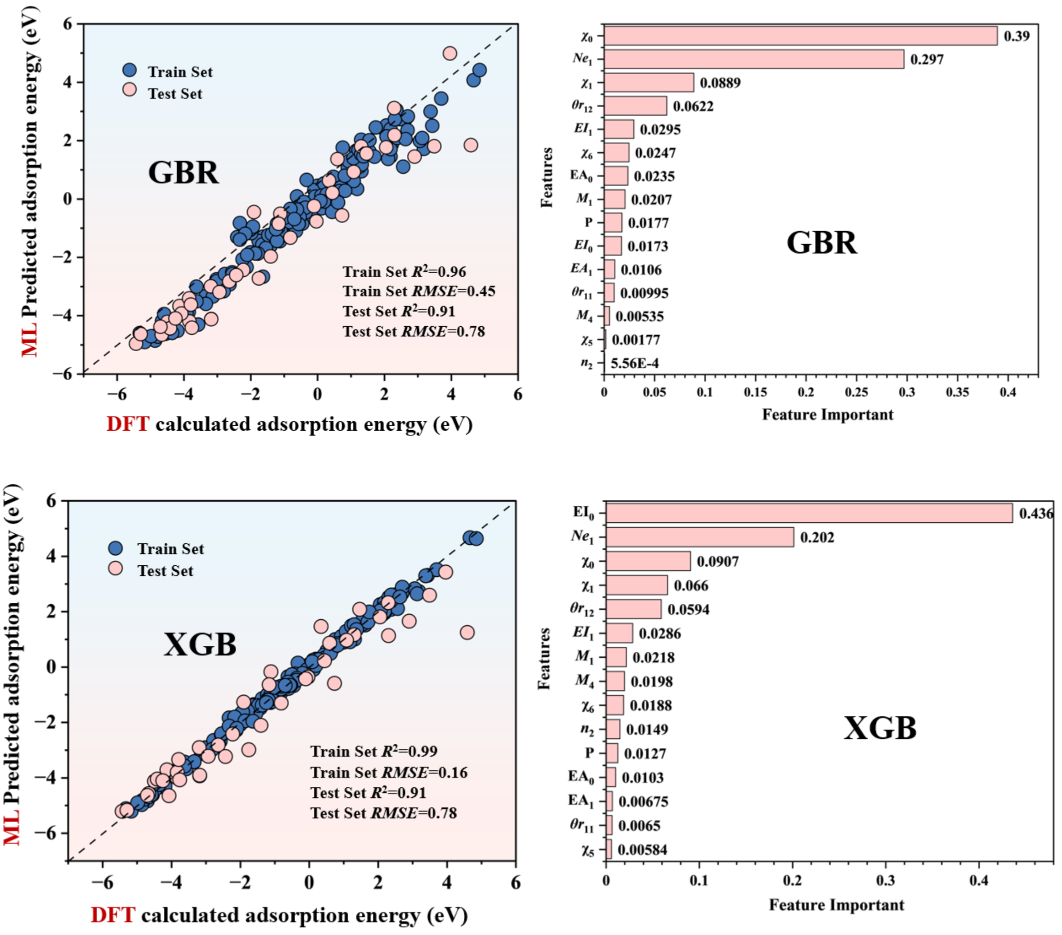


**Fig. S3** Fitting Accuracy and Feature Importance of GBR, XGB and GBR Algorithms Before Feature Engineering.

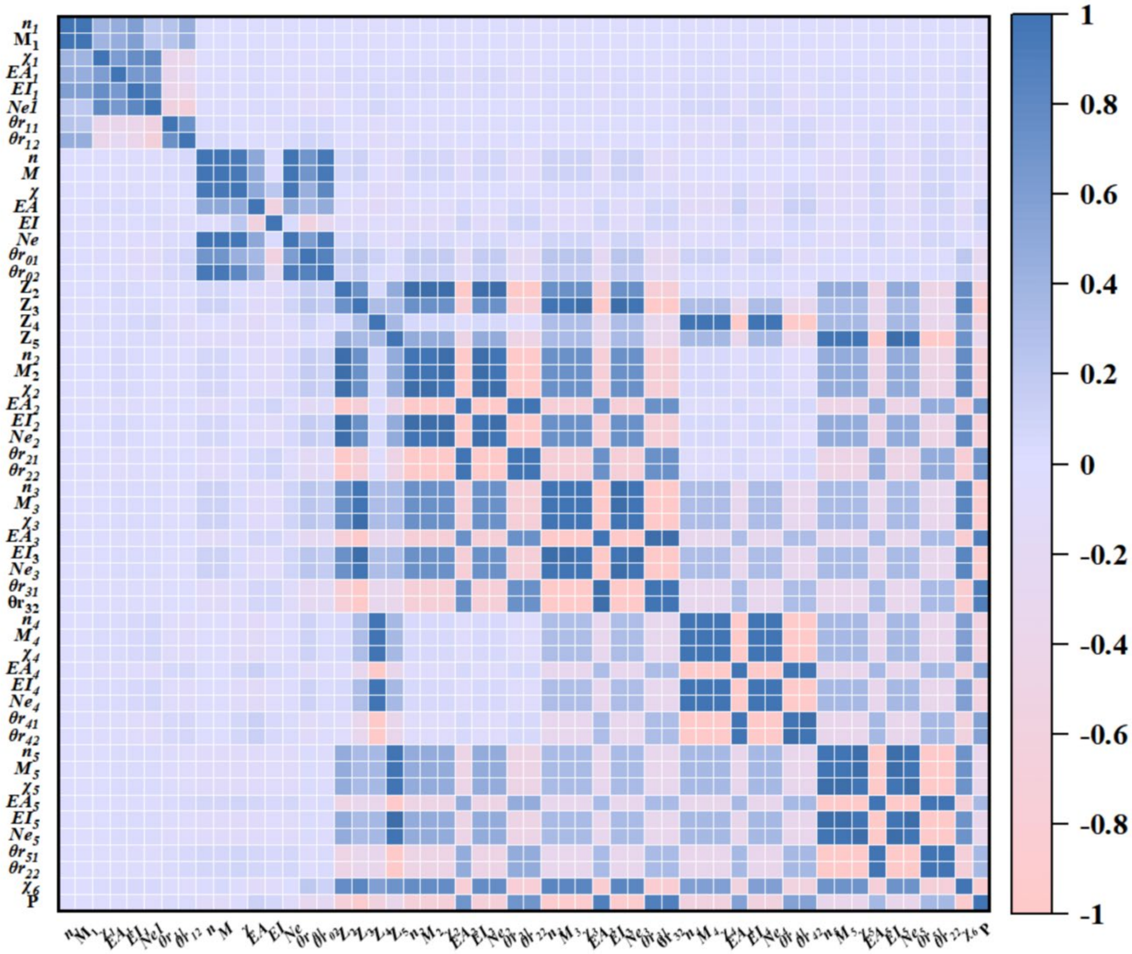


**Fig. S4** Correlation Heatmap of the Dataset Before Feature Processing**.**

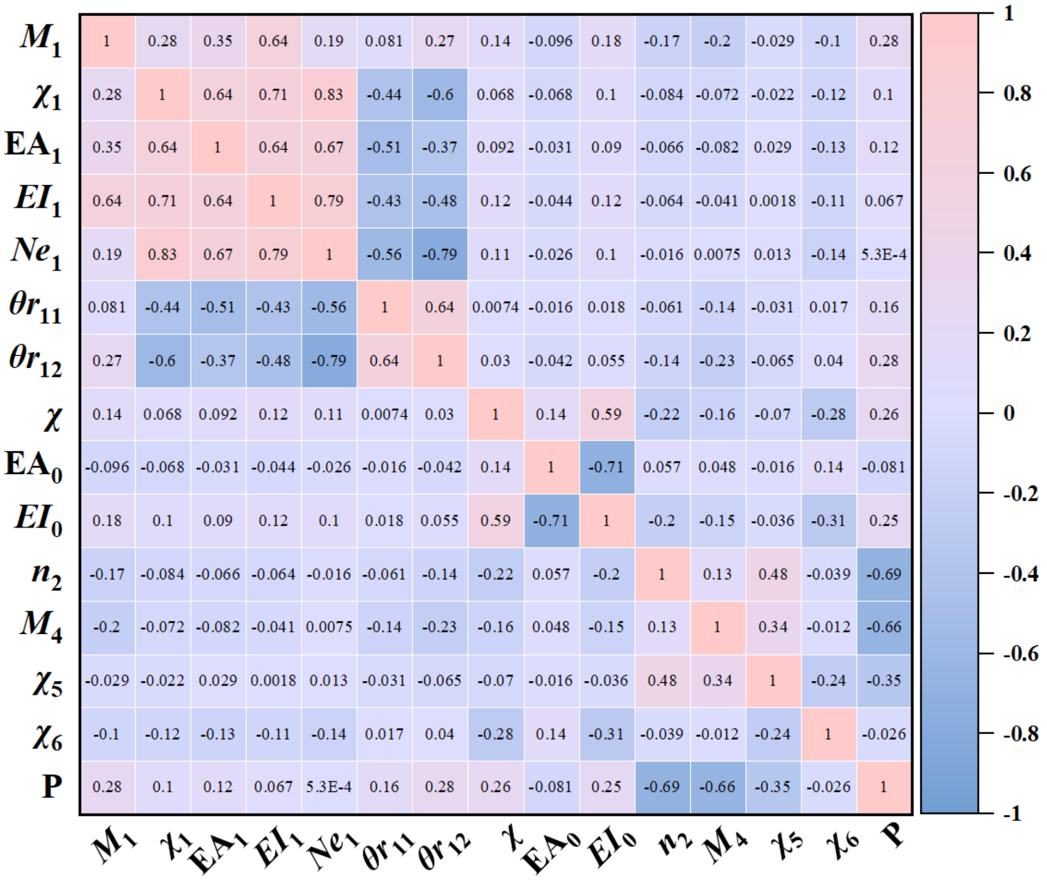


**Fig. S5** Correlation Heatmap of Feature-Processed Dataset.

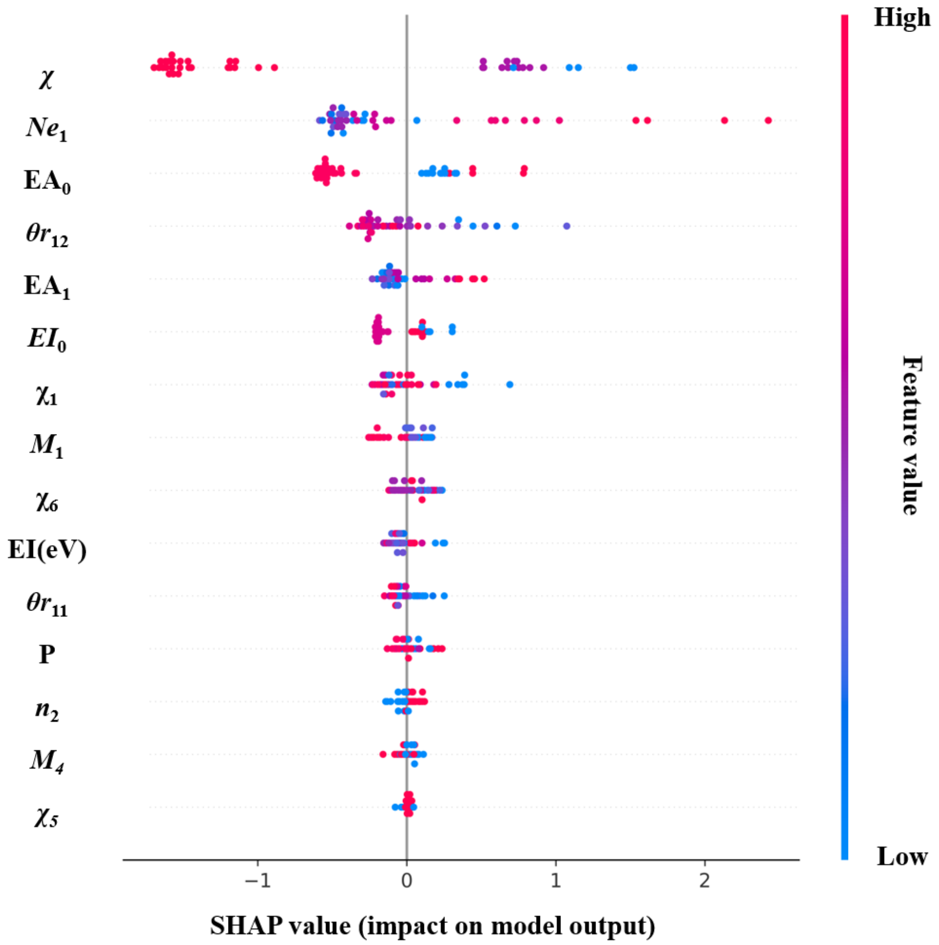


**Fig. S6** SHAP Analysis of Feature-Processed Ensemble Models.

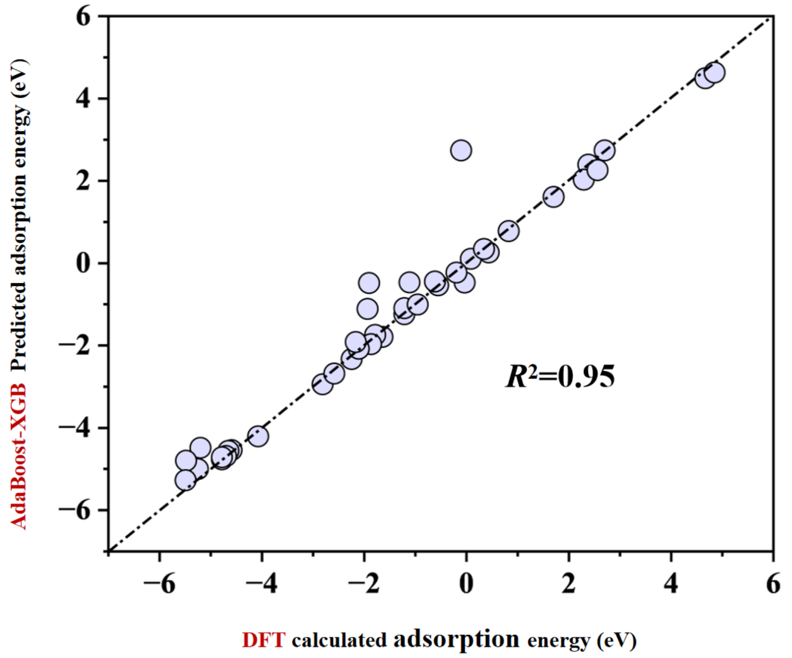


**Fig. S7** Plot of adsorption energies predicted by AdaBoost-GBR versus those calculated by DFT.

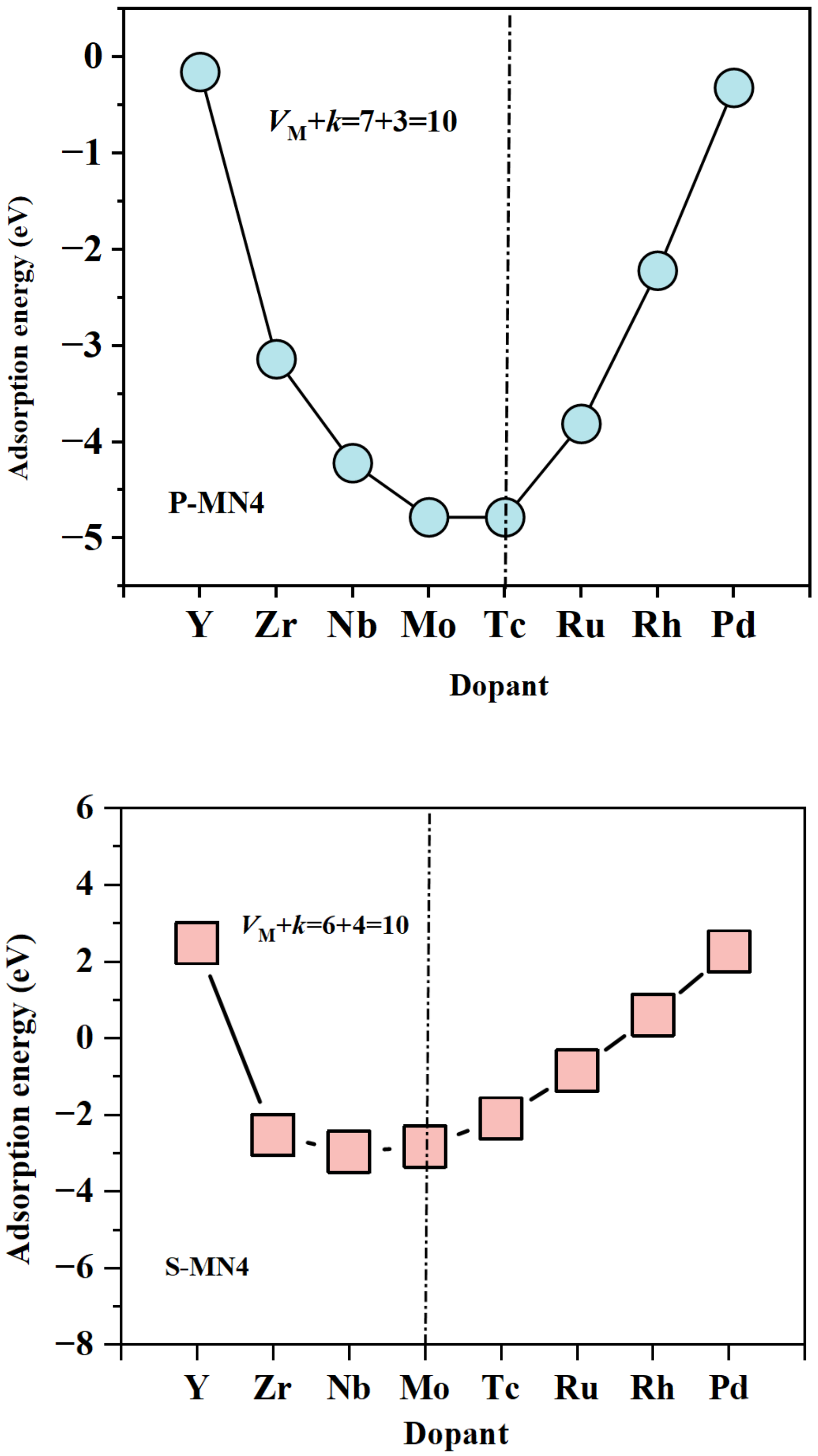


**Fig. S8** The variation law of adsorption energy of S and P atoms on MN4.

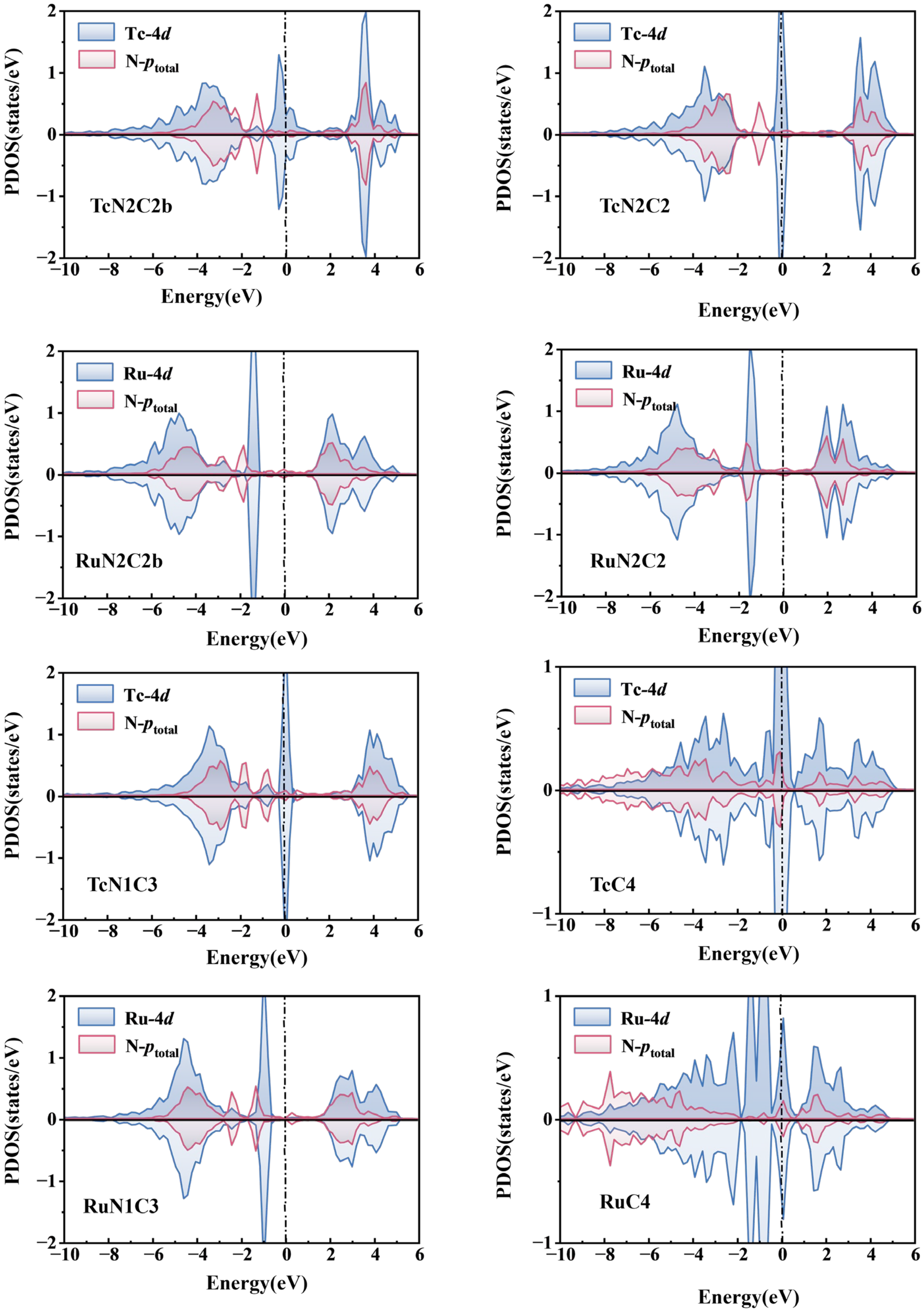


**Fig. 9** The PDOS of TcC4, RuC4, TcC3N1, RuC3N1, TcN2C2, RuN2C2, TcN2C2b and RuN2C2b for the adsorption of N.

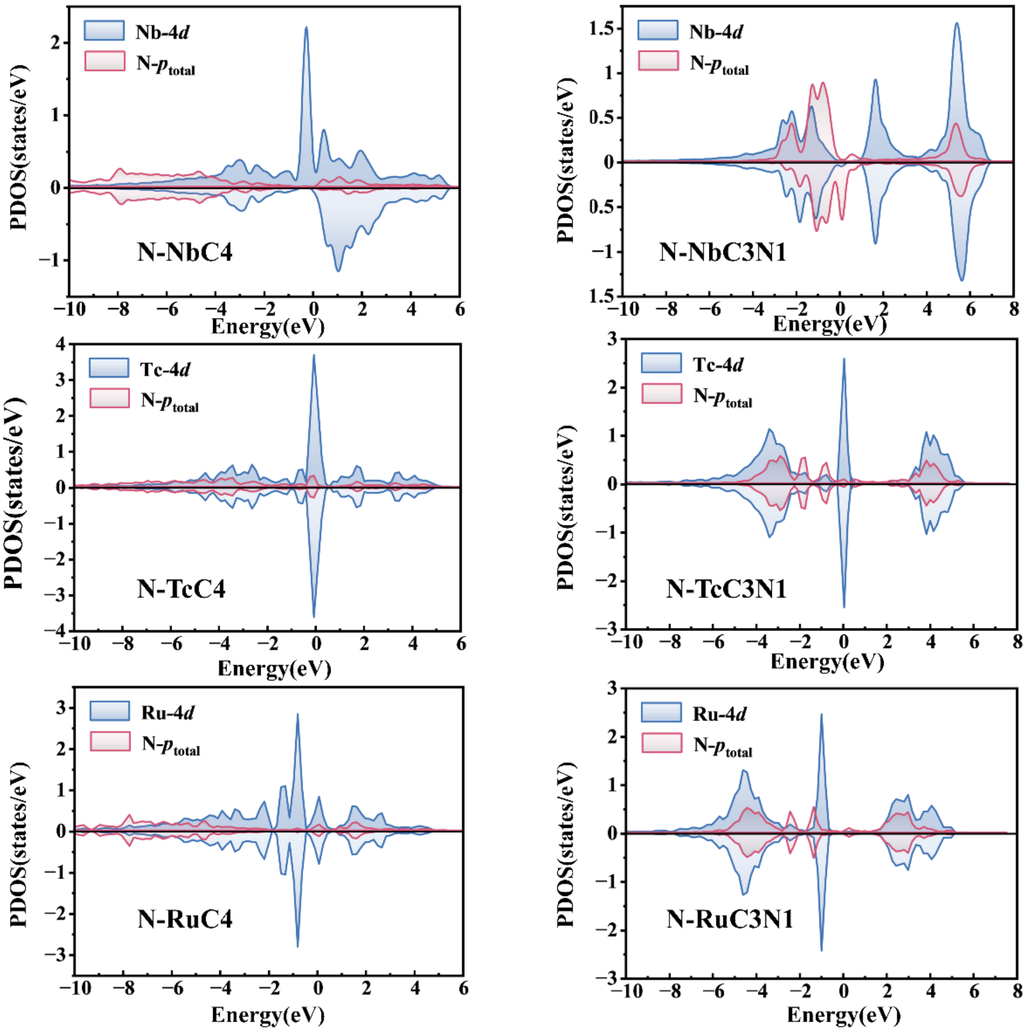


**Fig. S10** The PDOS of NbC4, TcC4, RuC4, NbC3N1, TcC3N1 and RuC3N1($\overline{\chi} < 2.8$) for the adsorption of N.

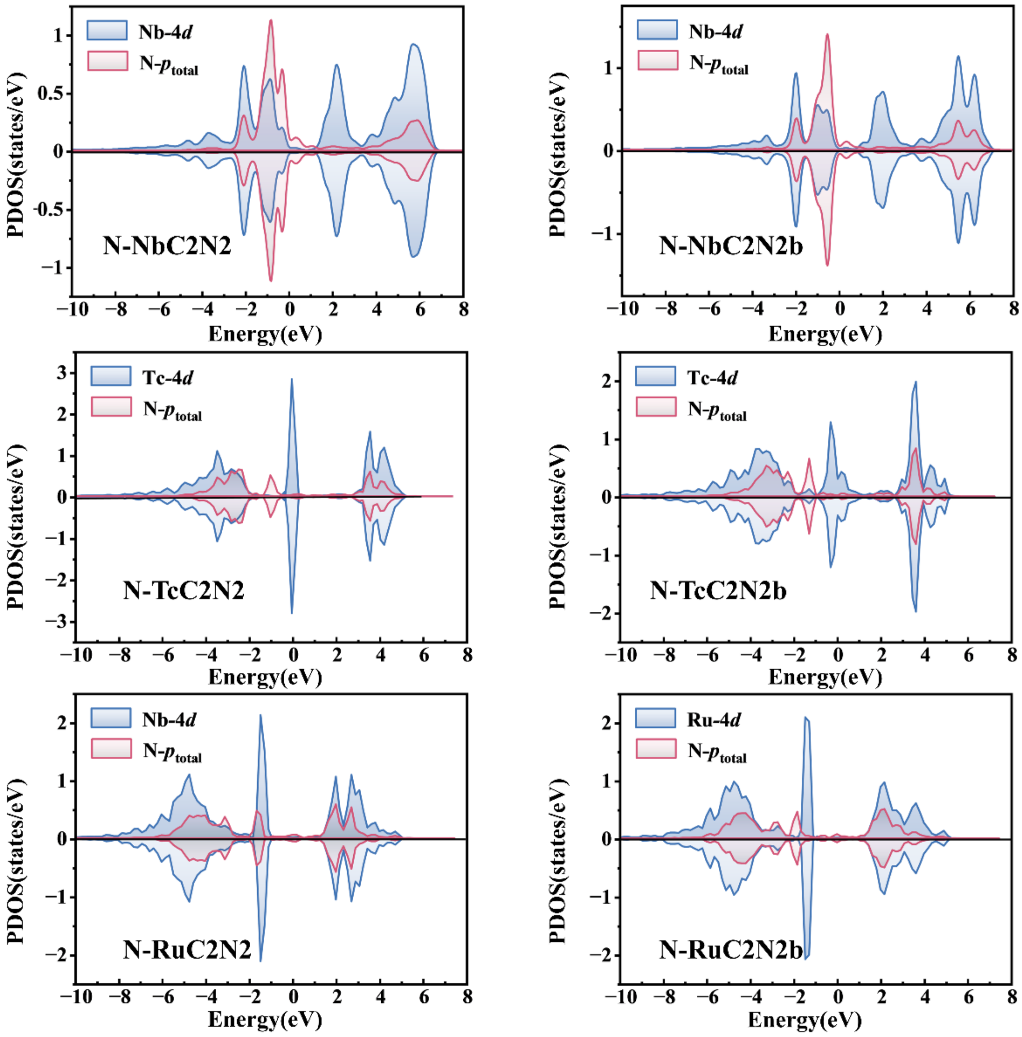


**Fig. S11** The PDOS of NbC2N2, TcC2N2, RuC2N2, NbC2N2b, TcC2N2b and RuC2N2b ($\overline{\chi} = 2.8$) for the adsorption of N.

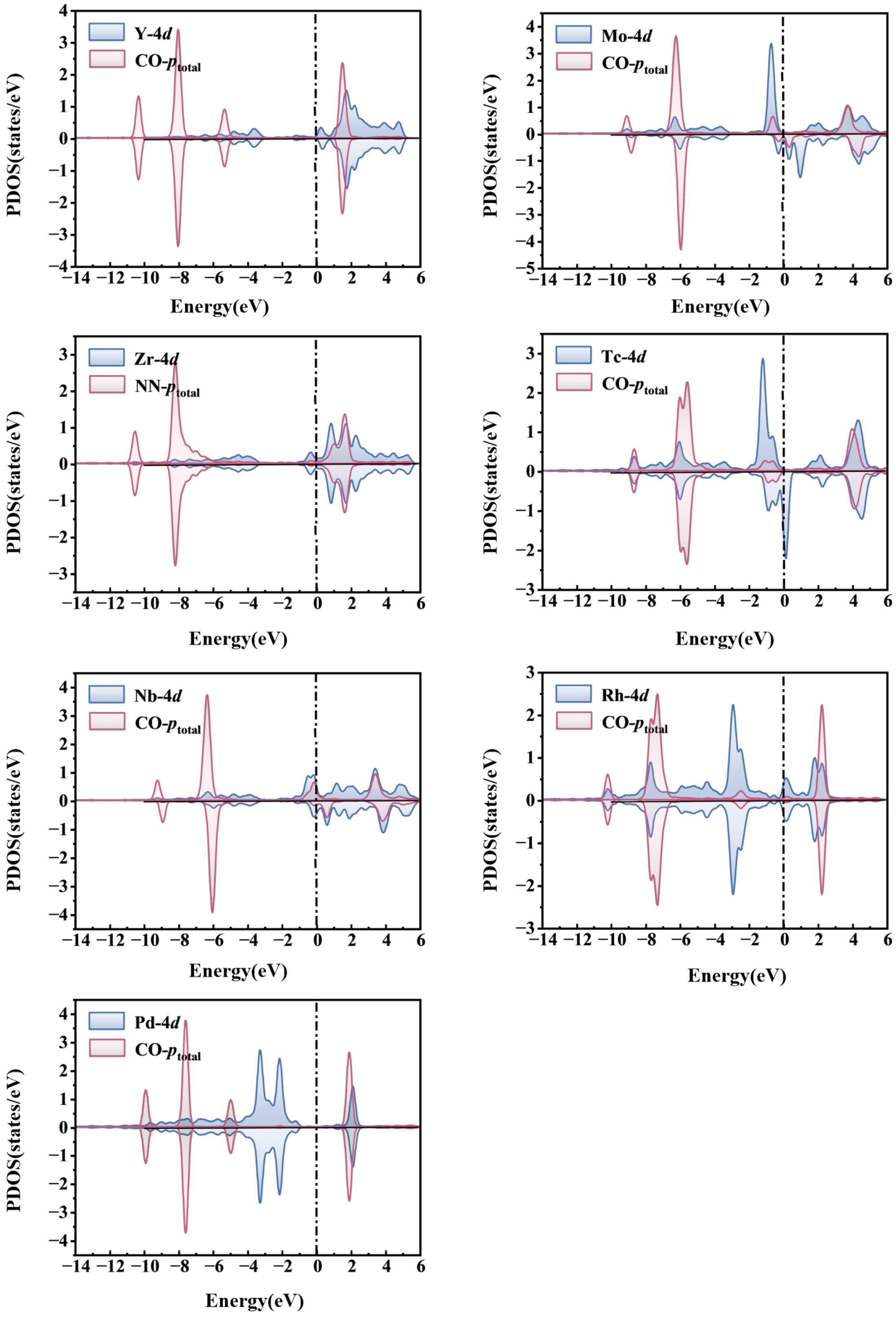


**Fig. S12** MN4 (M =4*d*) adsorbs PDOS of CO.

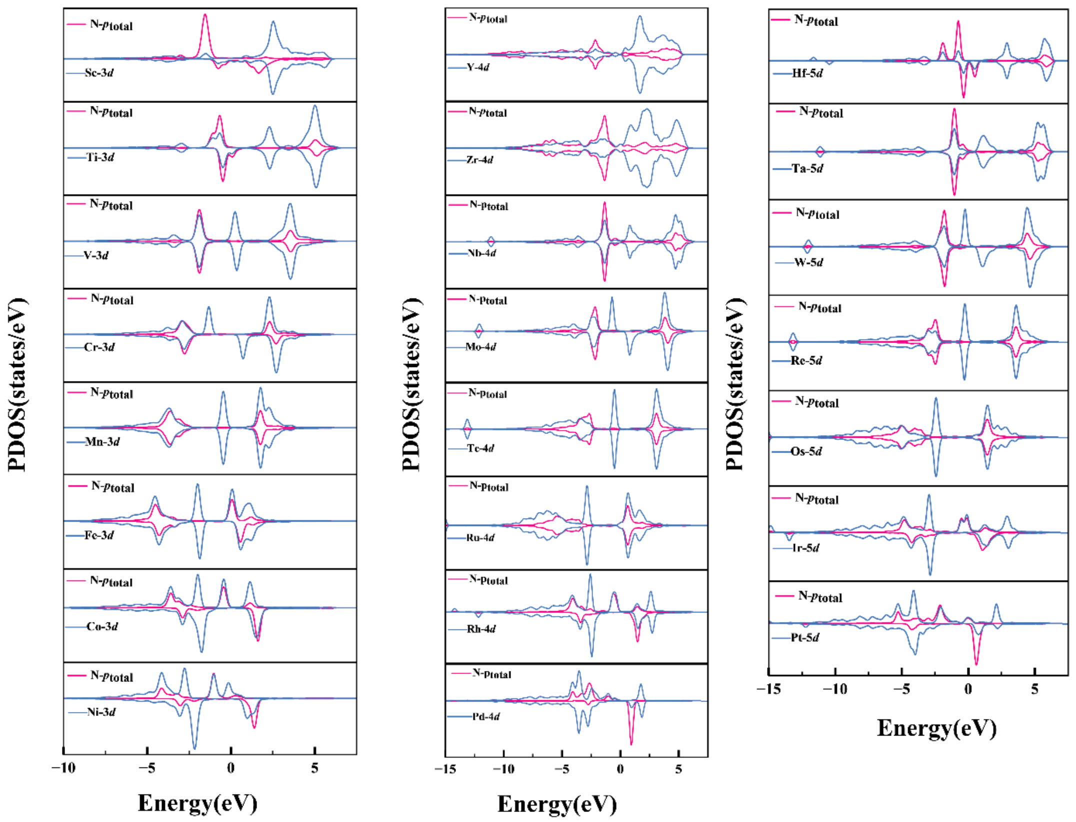


**Fig. S13** MN4 (M =3*d*, 4*d* and 5*d*) adsorbs PDOS of N.

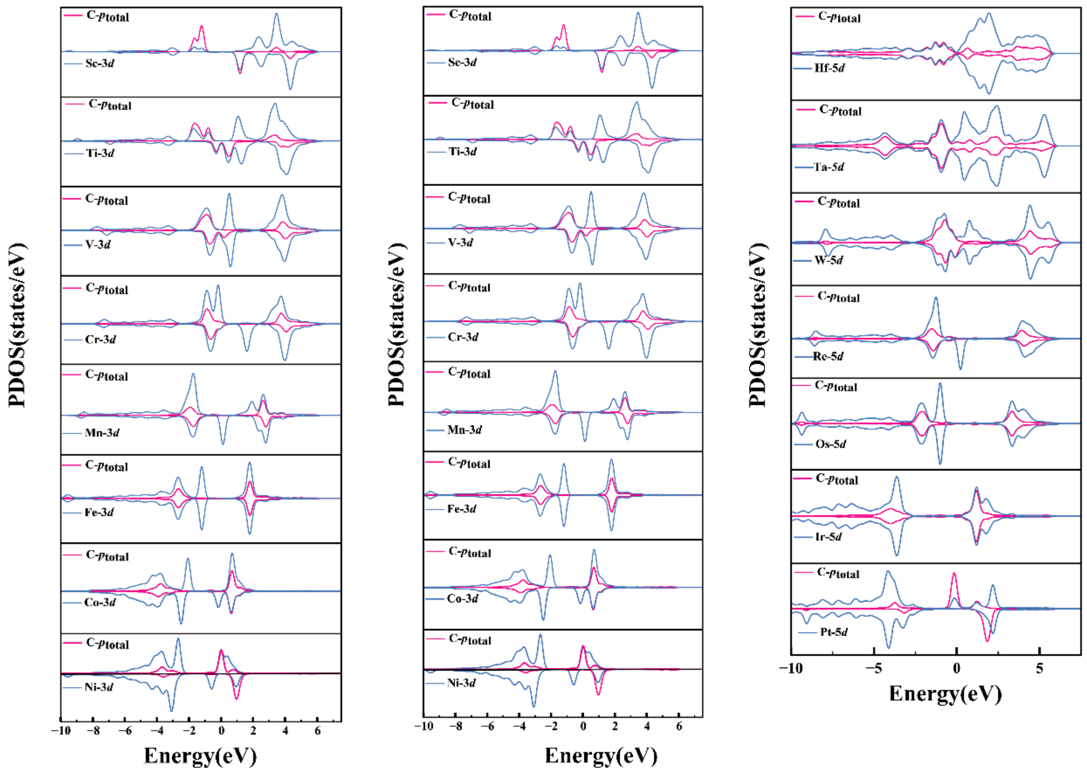


**Fig. S14** MN4 (M =3*d*, 4*d* and 5*d*) adsorbs PDOS of C.

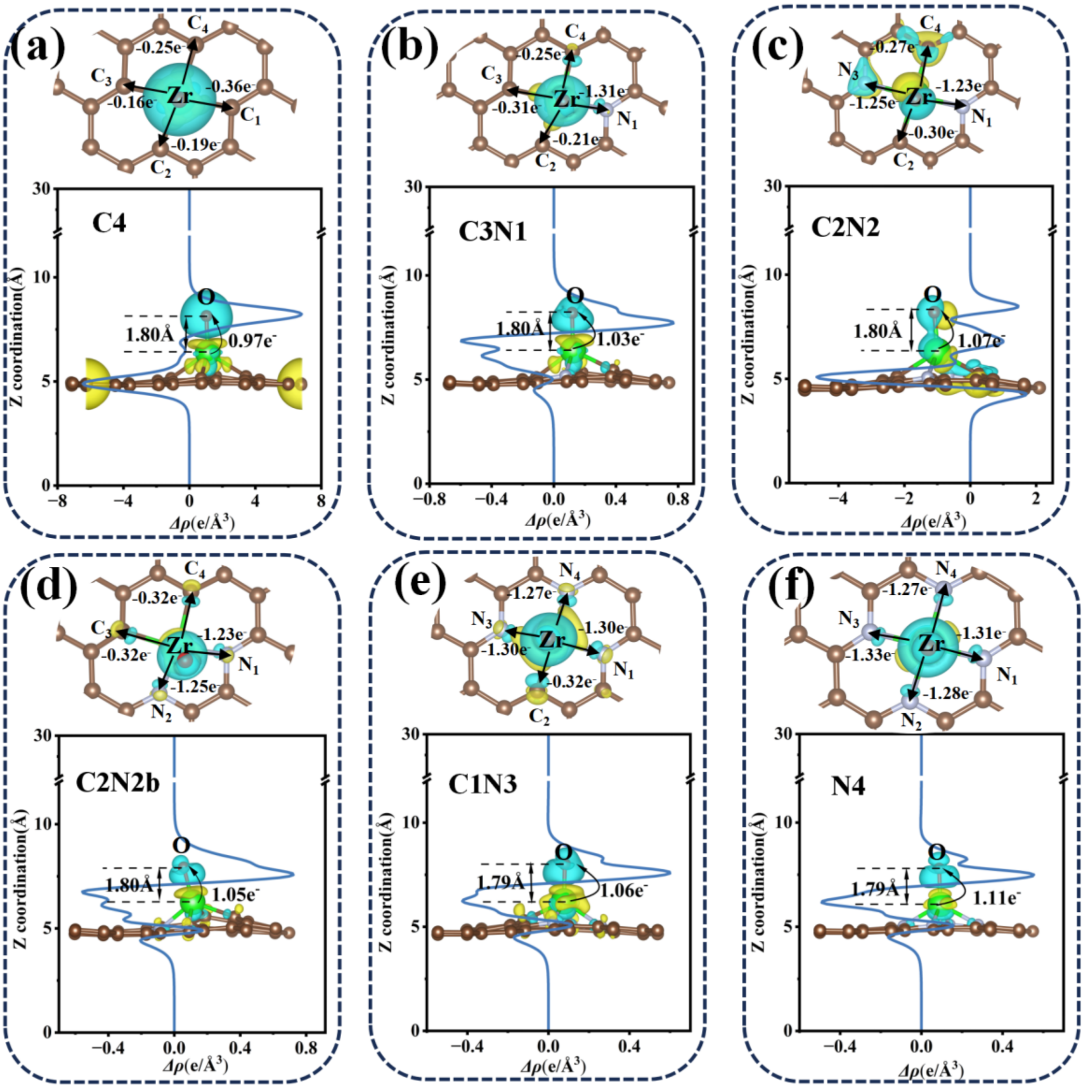

**Fig. S15** The three-dimensional charge density difference of Zr-doped MC4, MN1C3, MN2C2, MN2C2b, MN3C1 and MN4 adsorbing O.

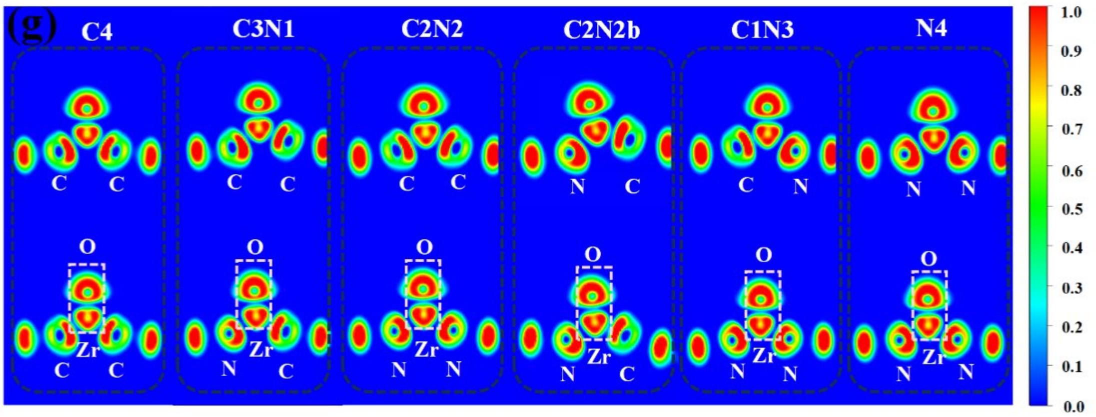

**Fig. S16** The ELF of Zr-doped MC4, MN1C3, MN2C2, MN2C2b, MN3C1 and MN4 structures adsorbing O.

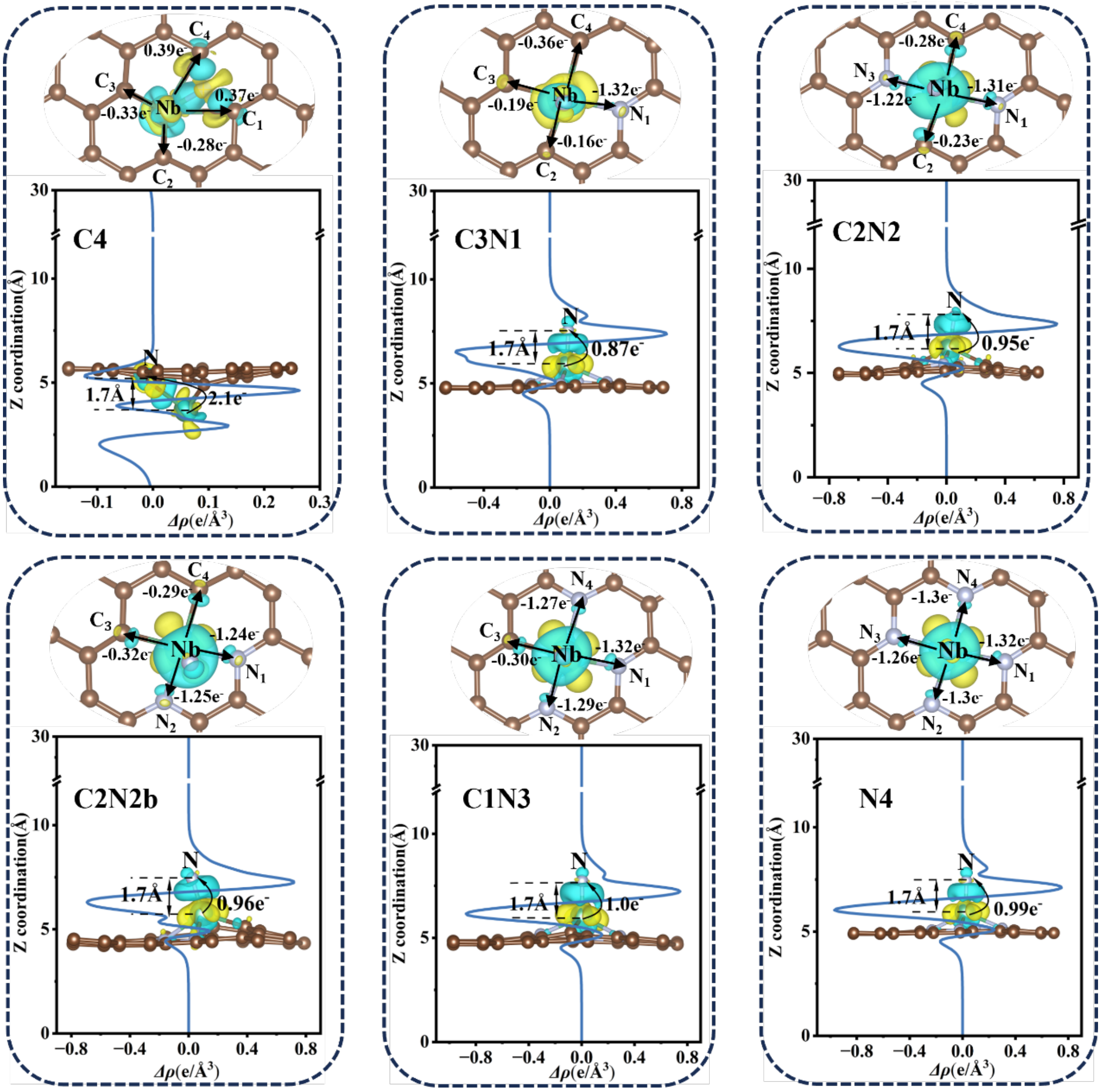

**Fig. S17** The three-dimensional charge density difference of Nb-doped MC4, MN1C3, MN2C2, MN2C2b, MN3C1 and MN4 adsorbing N.

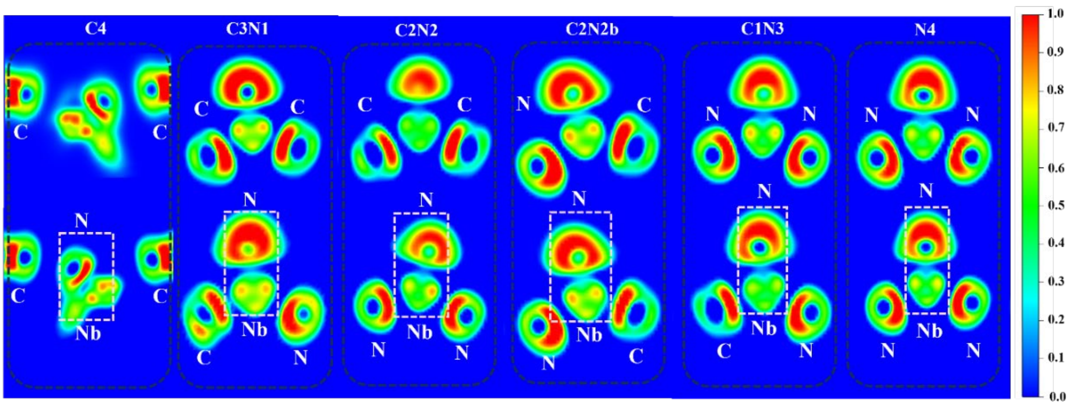

Fig. S18 The ELF of Nb-doped MC4, MN1C3, MN2C2, MN2C2b, MN3C1 and MN4 structures adsorbing N.

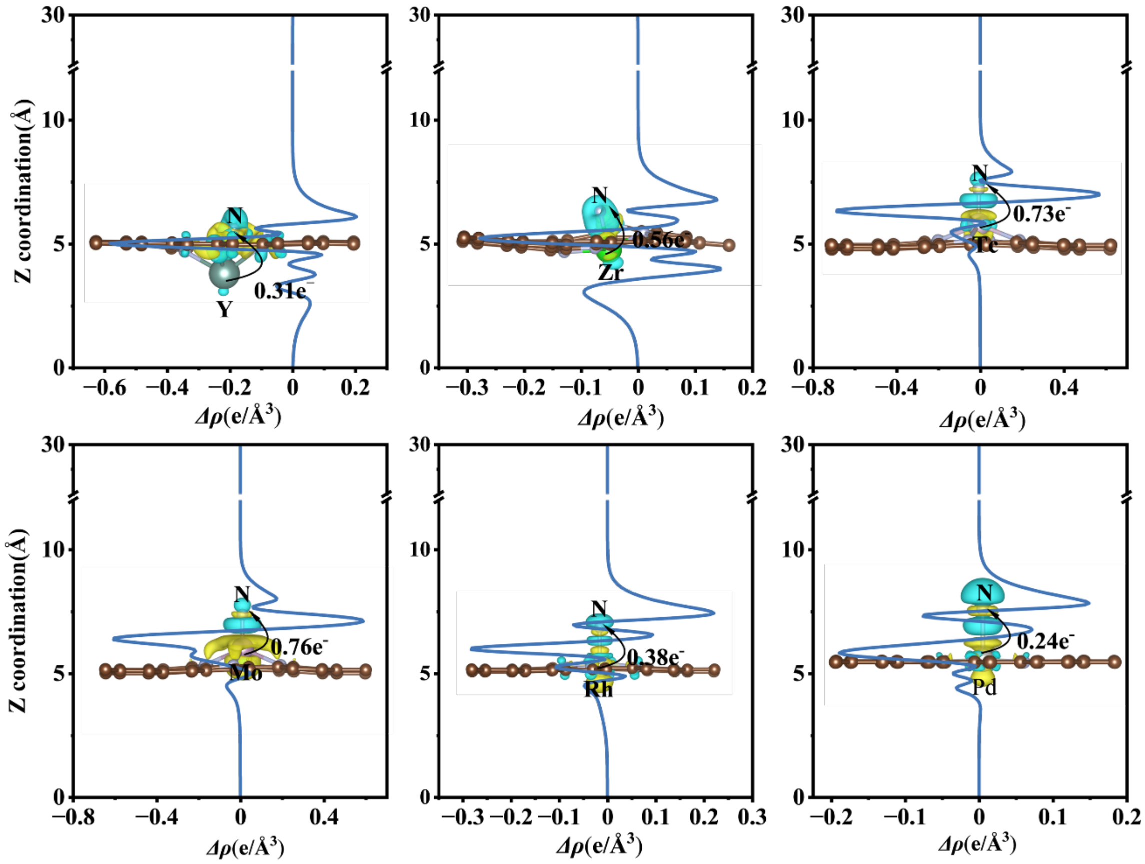


**Fig. S19** Three-dimensional charge density difference of MN4(M=4*d*) adsorption of N.

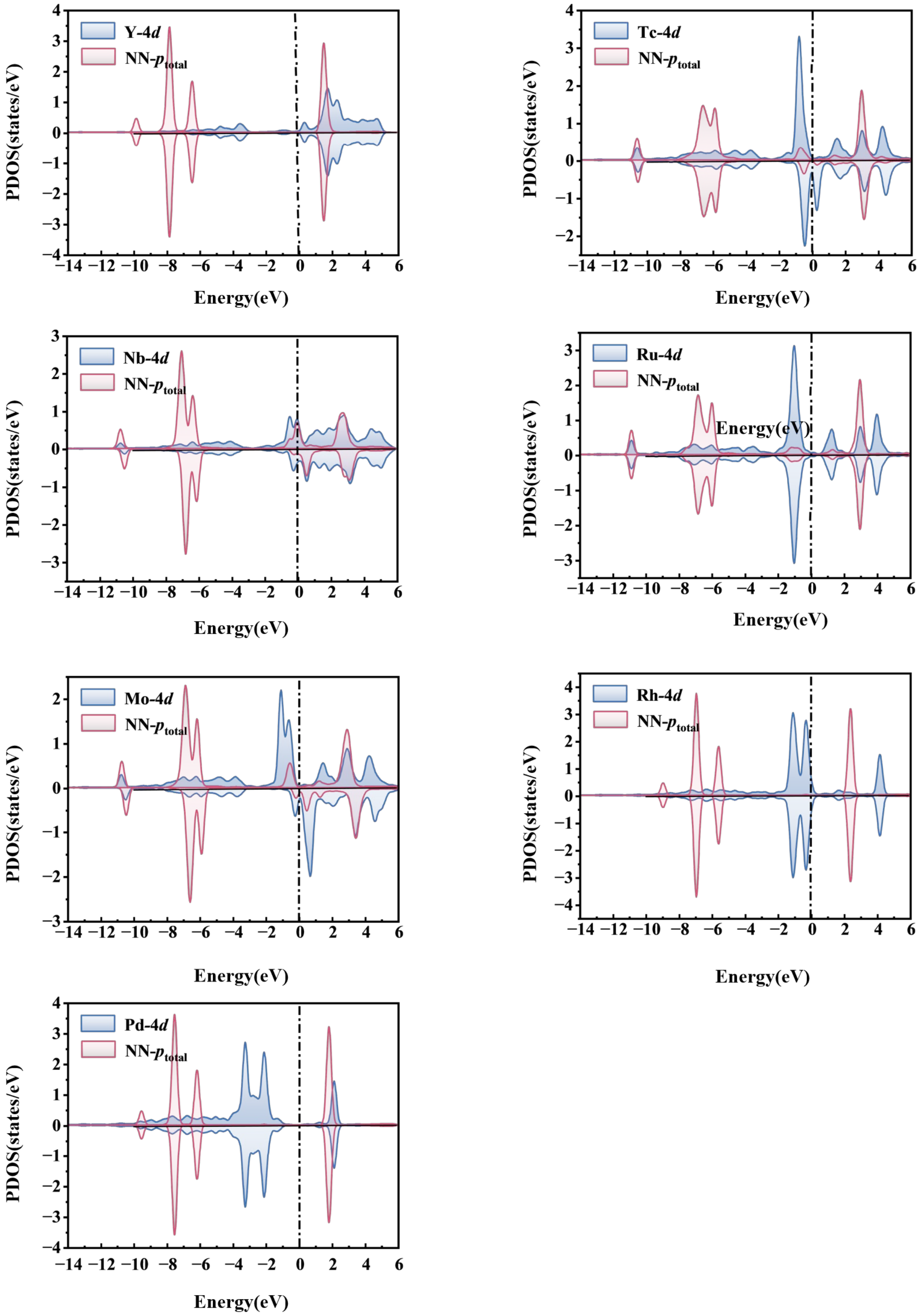


**Fig. S20** MN4 (M =4*d*) adsorbs PDOS of NN.

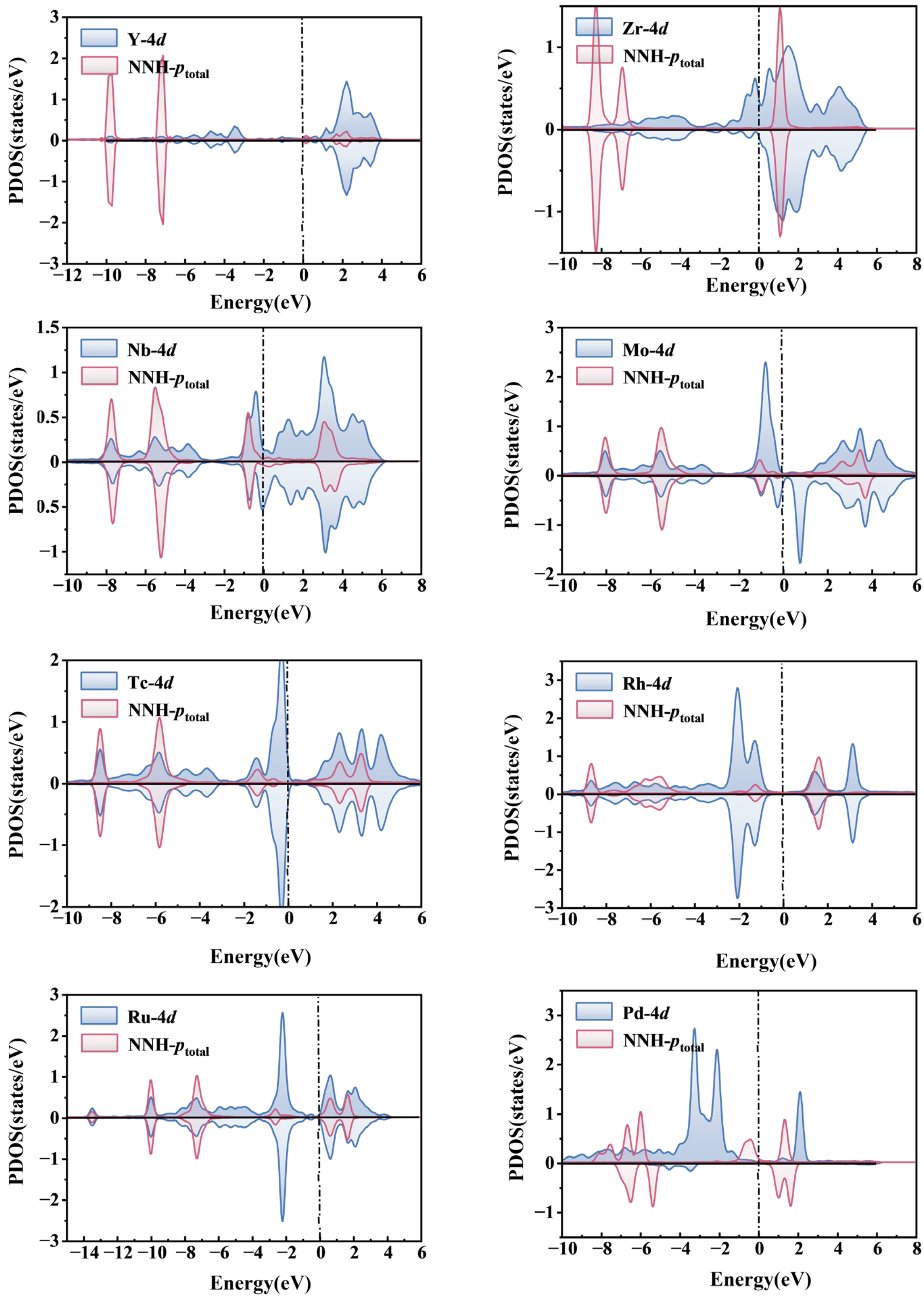


**Fig. S21** MN4 (M =4*d*) adsorbs PDOS of NNH.

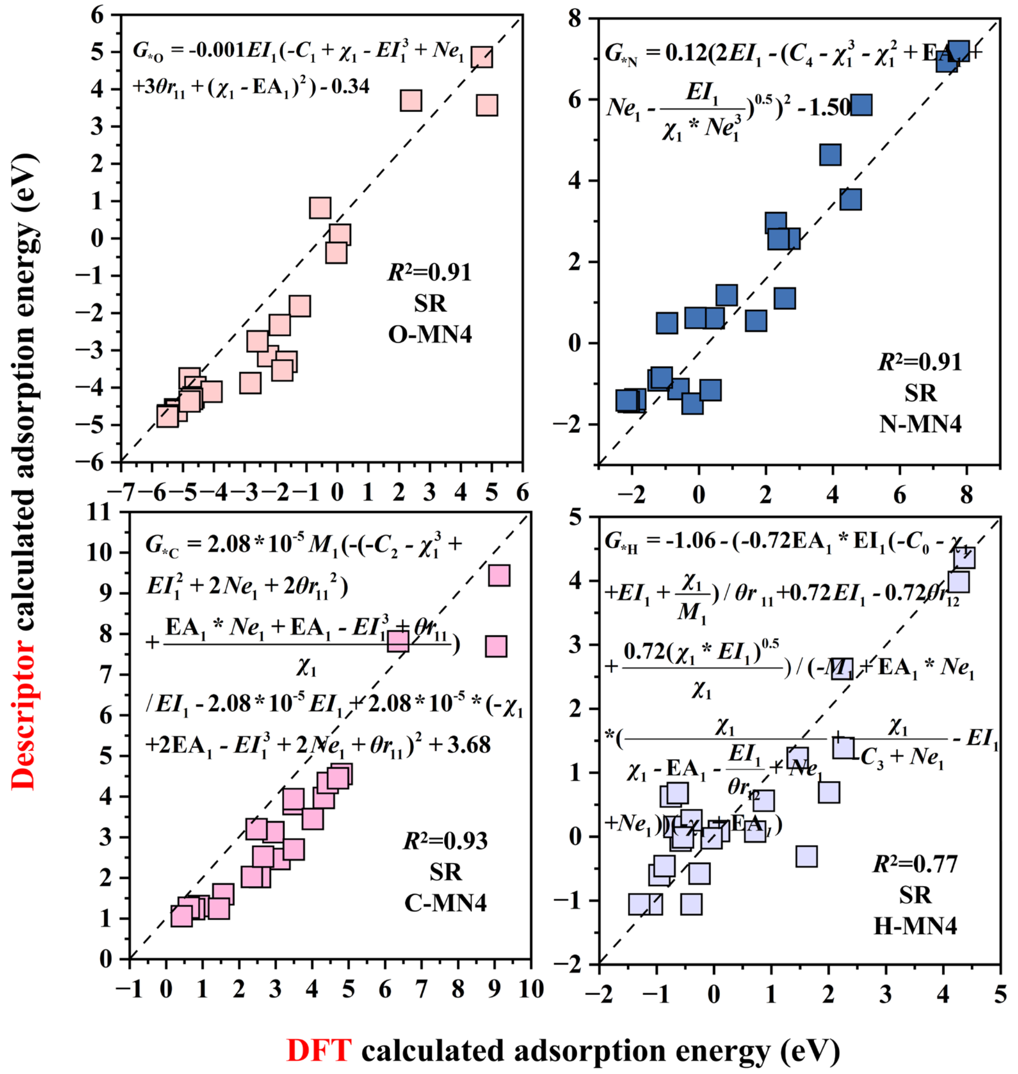


**Fig. S22** Comparison of SR descriptor predictions for MN4(M=3*d*,4*d* and 5*d*) adsorption intermediates (O, N, C and H) with DFT calculation results.

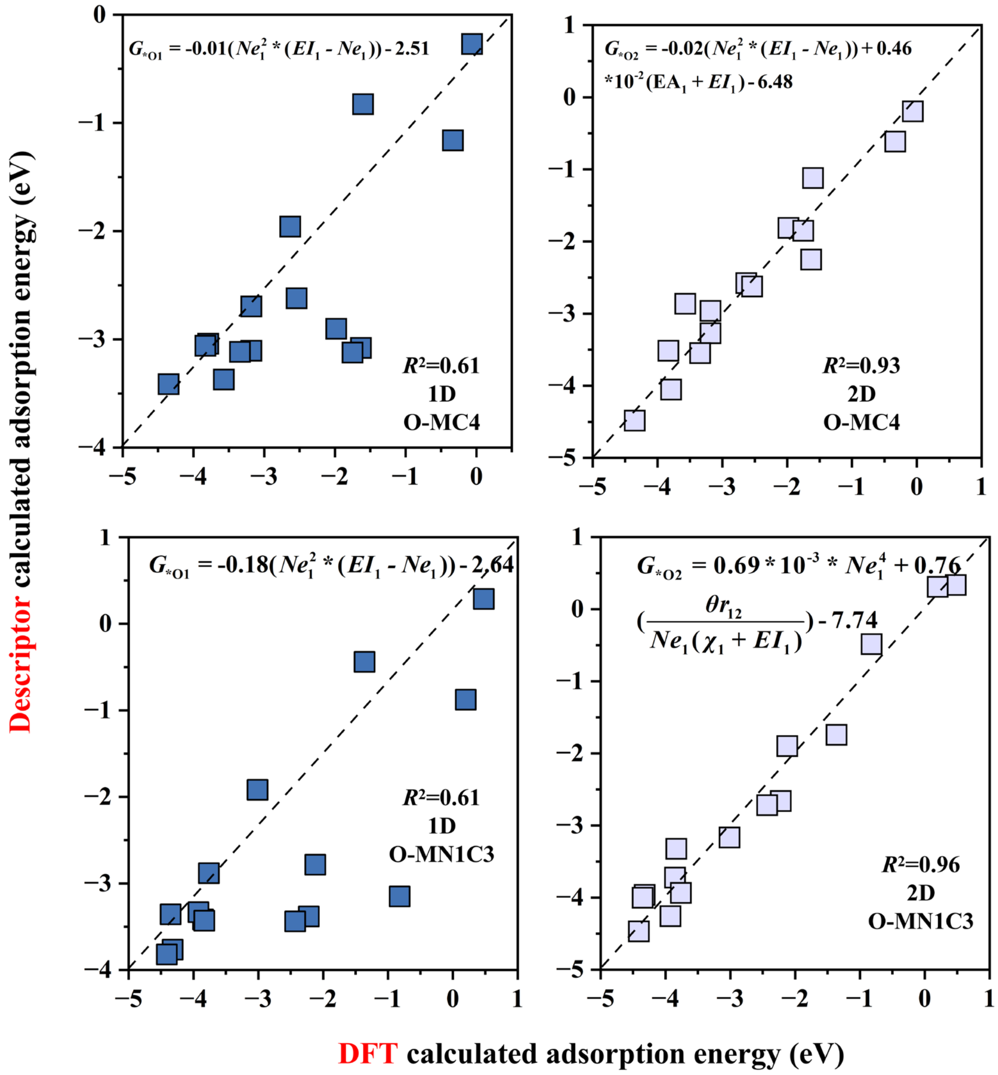

$G_{*O1} = -0.01(Ne_1^2 * (EI_1 - Ne_1)) - 2.51$
$R^2$=0.61
1D
O-MC4
$G_{*O2} = -0.02(Ne_1^2 * (EI_1 - Ne_1)) + 0.46 * 10^{-2}(EA_1 + EI_1) - 6.48$
$R^2$=0.93
2D
O-MC4
$G_{*O1} = -0.18(Ne_1^2 * (EI_1 - Ne_1)) - 2.64$
$R^2$=0.61
1D
O-MN1C3
$G_{*O2} = 0.69 * 10^{-3} * Ne_1^4 + 0.76(\frac{\theta r_{12}}{Ne_1(\chi_1 + EI_1)}) - 7.74$
$R^2$=0.96
2D
O-MN1C3
Descriptor calculated adsorption energy (eV)
DFT calculated adsorption energy (eV)

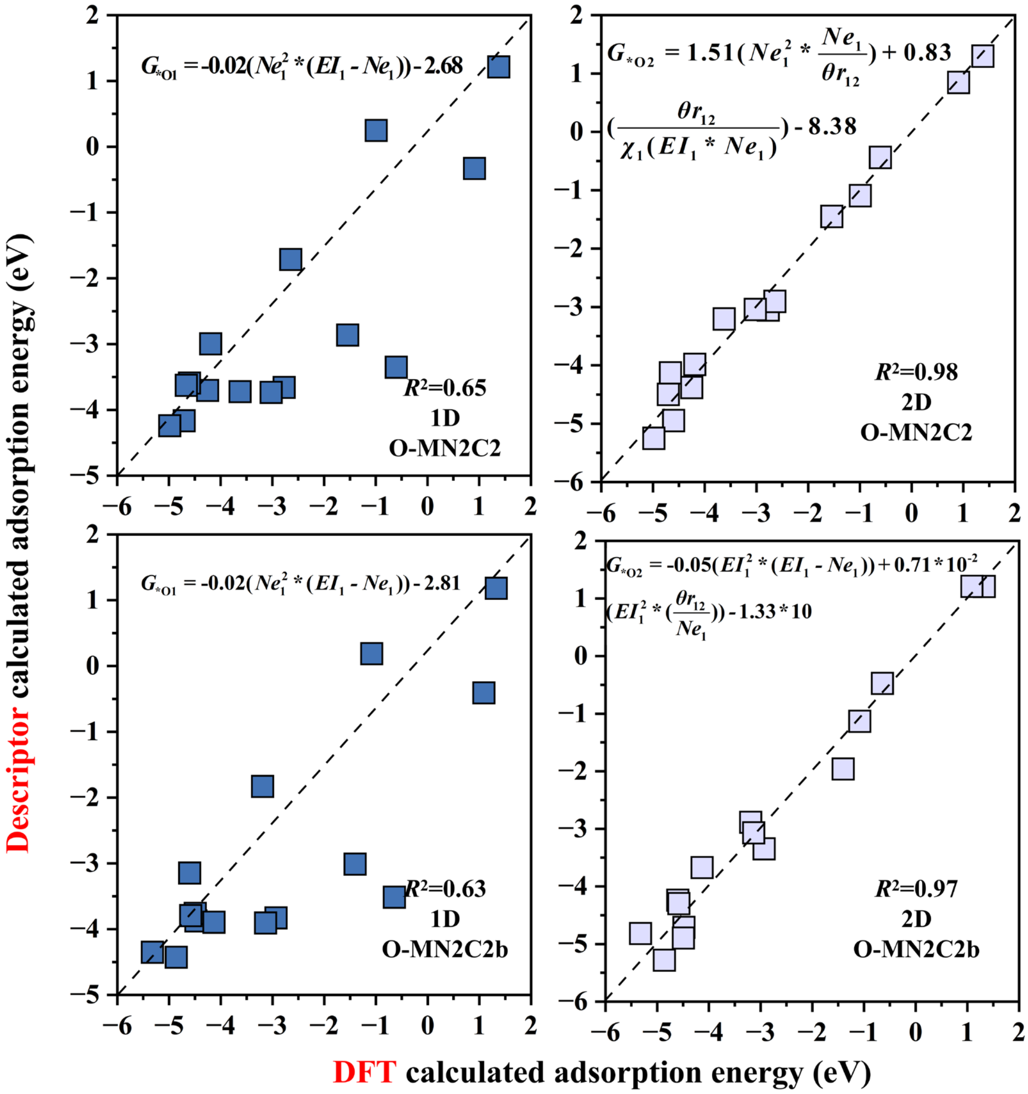

$G_{*O1} = -0.02(Ne_1^2 * (EI_1 - Ne_1)) - 2.68$
$R^2$=0.65
1D
O-MN2C2
$G_{*O2} = 1.51(Ne_1^2 * \frac{Ne_1}{\theta r_{12}}) + 0.83 (\frac{\theta r_{12}}{\chi_1(EI_1 * Ne_1)}) - 8.38$
$R^2$=0.98
2D
O-MN2C2
$G_{*O1} = -0.02(Ne_1^2 * (EI_1 - Ne_1)) - 2.81$
$R^2$=0.63
1D
O-MN2C2b
$G_{*O2} = -0.05(EI_1^2 * (EI_1 - Ne_1)) + 0.71 * 10^{-2} (EI_1^2 * (\frac{\theta r_{12}}{Ne_1})) - 1.33 * 10$
$R^2$=0.97
2D
O-MN2C2b
Descriptor calculated adsorption energy (eV)
DFT calculated adsorption energy (eV)

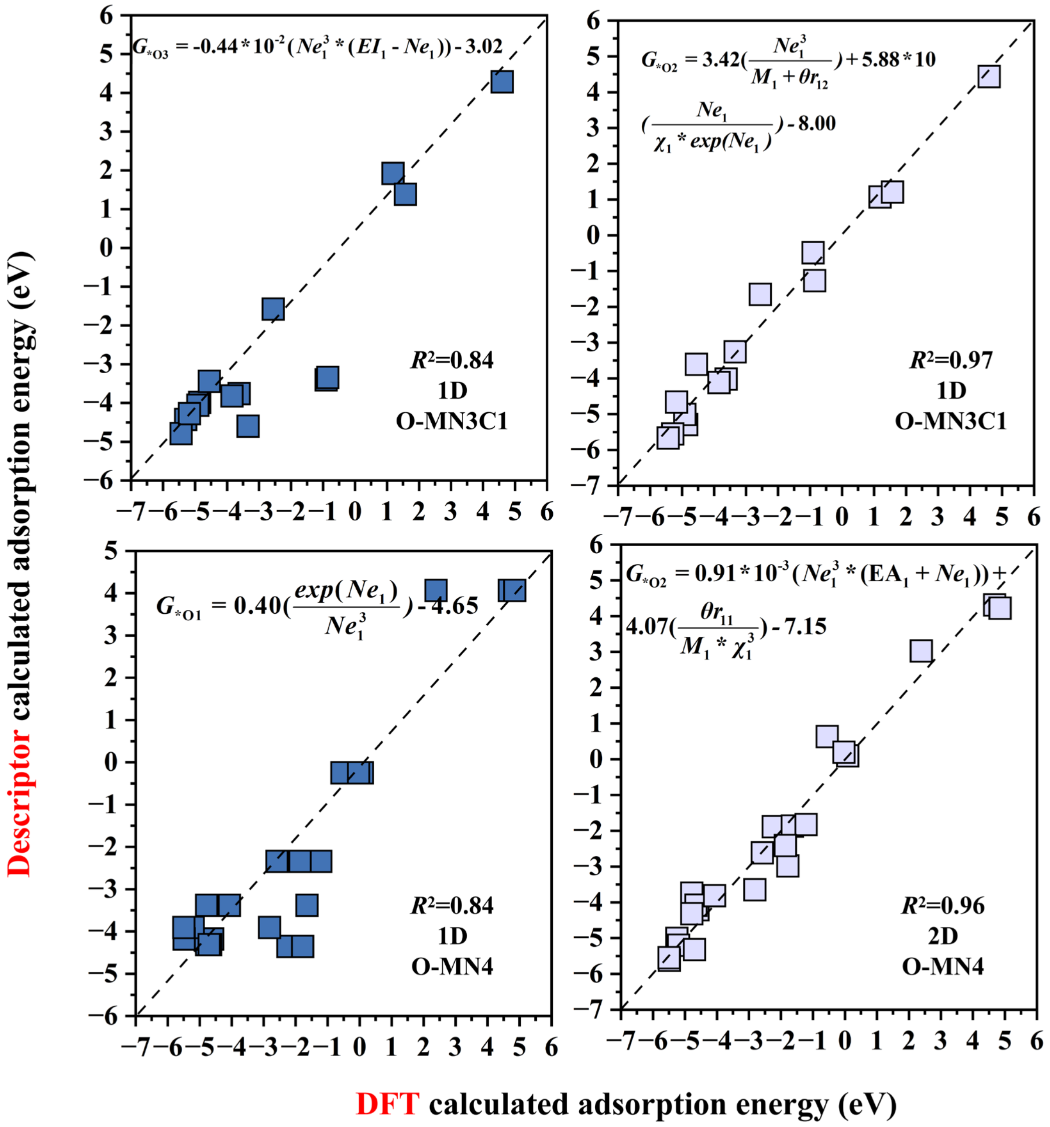


**Fig. S23** Comparison of SISSO descriptor predictions (1D and 2D) for MC4, MN1C3, MN2C2, MN2C2b, MN3C1 and MN4 (M = 4d and 5d) adsorption intermediate O with DFT calculation results.

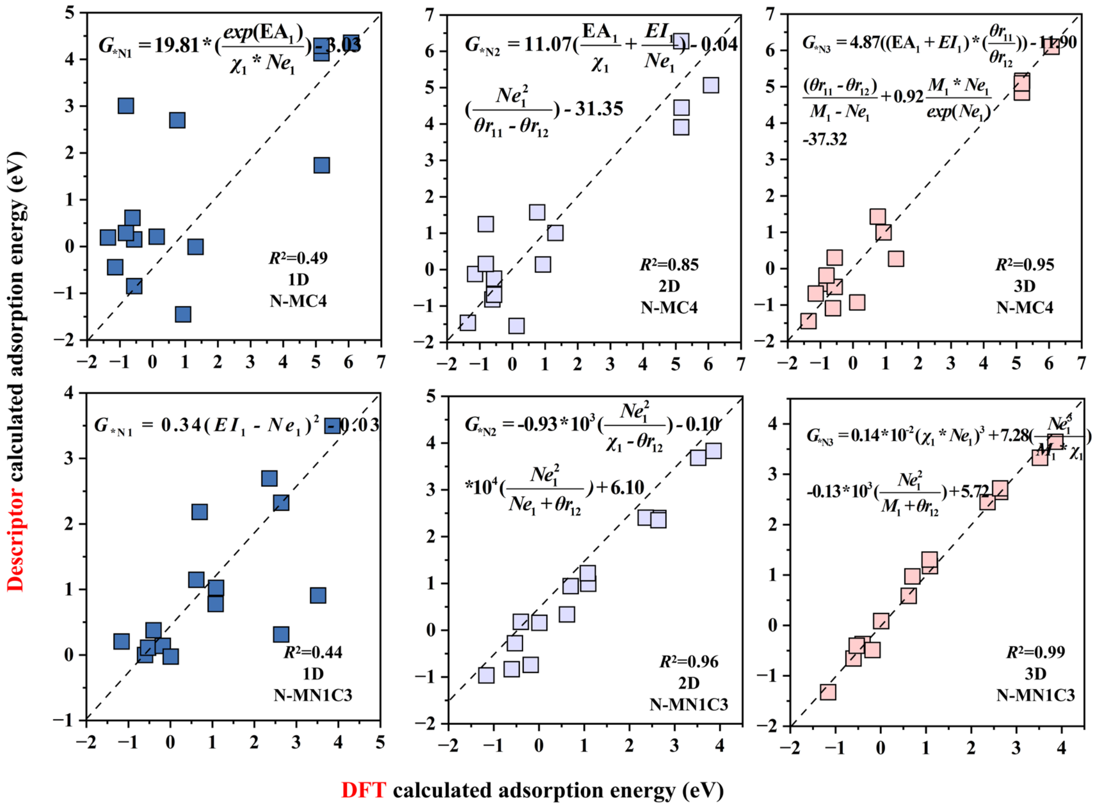
Descriptor calculated adsorption energy (eV)
DFT calculated adsorption energy (eV)
R²=0.49
1D
N-MC4
R²=0.85
2D
N-MC4
R²=0.95
3D
N-MC4
R²=0.44
1D
N-MN1C3
R²=0.96
2D
N-MN1C3
R²=0.99
3D
N-MN1C3

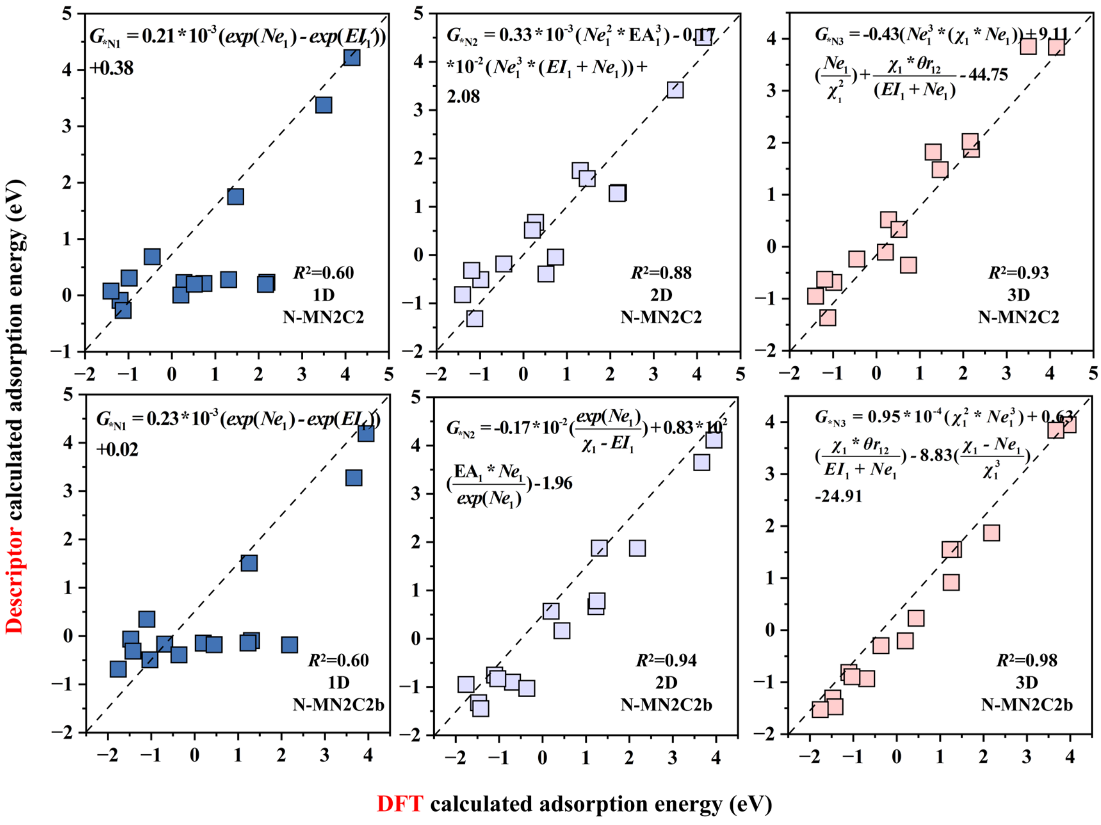
Descriptor calculated adsorption energy (eV)
DFT calculated adsorption energy (eV)
R²=0.60
1D
N-MN2C2
R²=0.88
2D
N-MN2C2
R²=0.93
3D
N-MN2C2
R²=0.60
1D
N-MN2C2b
R²=0.94
2D
N-MN2C2b
R²=0.98
3D
N-MN2C2b

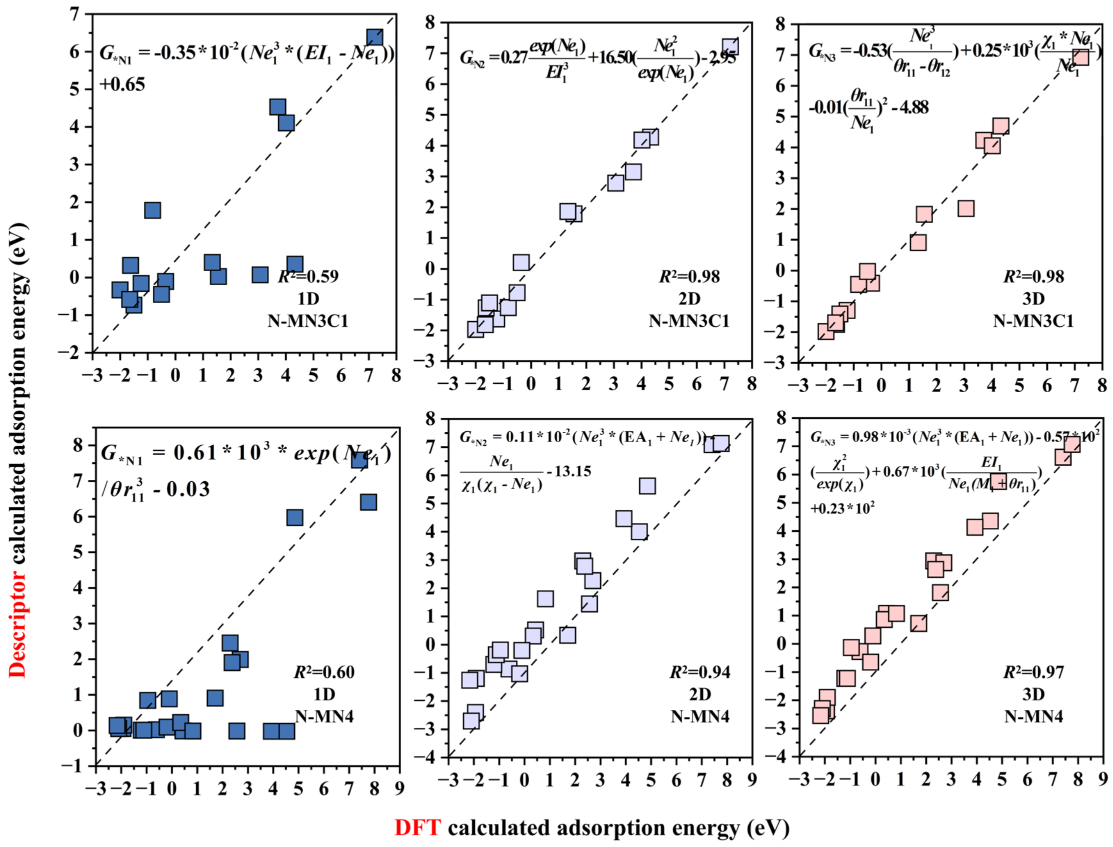


**Fig. S24** Comparison of SISSO descriptor predictions (1D, 2D and 3D) for MC4, MC3N1, MC2N2, MC2N2b, MC1N3 and MN4 (M = 4d and 5d) adsorption intermediate N with DFT calculation results.

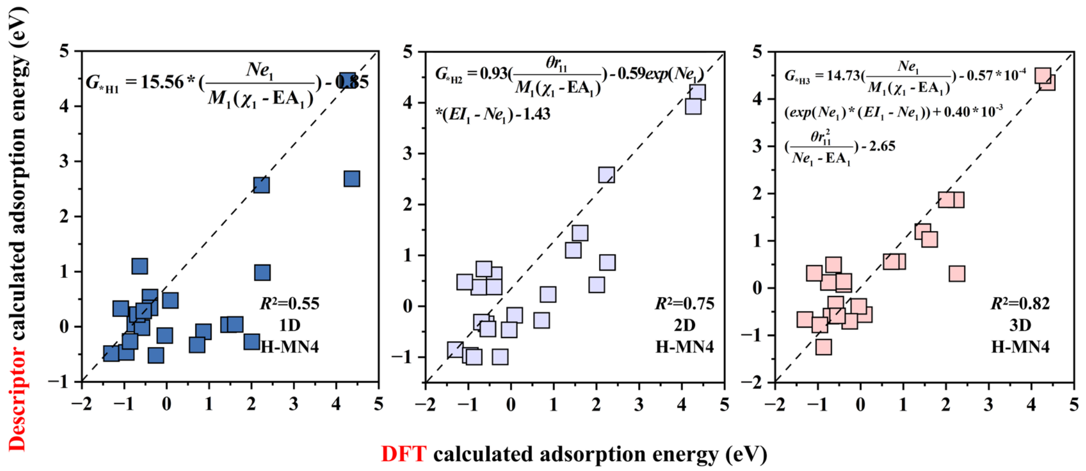


**Fig. S25** Comparison of SISSO descriptor predictions (1D, 2D and 3D) for MN4 (M = 3d, 4d and 5d) adsorption intermediate H with DFT calculation results.

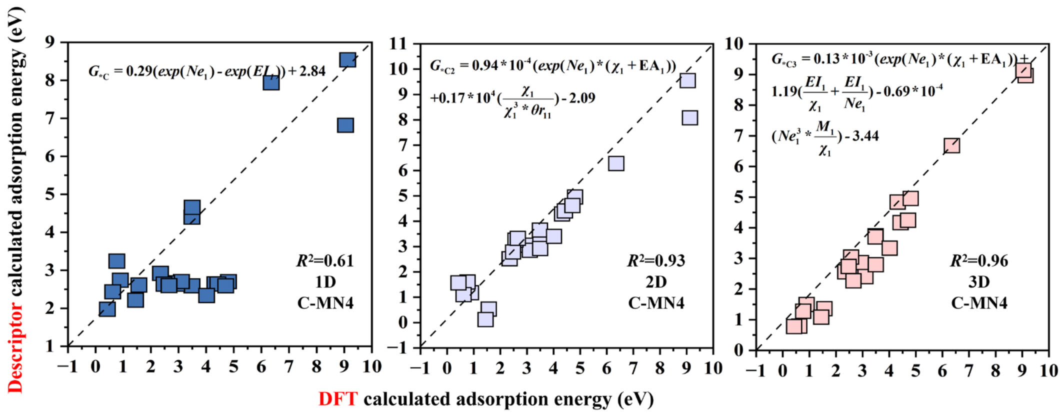


**Fig. S26** Comparison of SISSO descriptor predictions (1D, 2D and 3D) for MN4 (M = 3*d*, 4*d* and 5*d*) adsorption intermediate C with DFT calculation results.

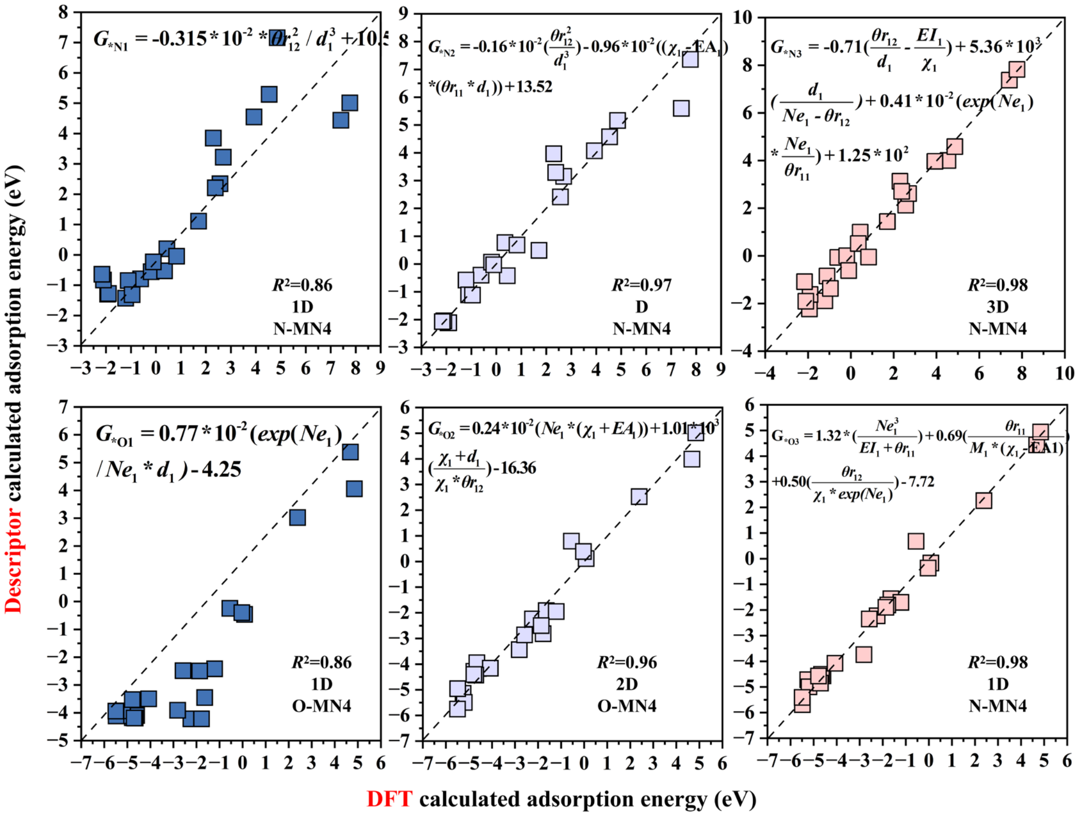


**Fig. S27** Comparison of SISSO descriptors (1D, 2D and 3D) with the introduction of bond length ($d_1$) for predicting MN4 (M = 3*d*, 4*d* and 5*d*) adsorption intermediate O and N with DFT calculation results.

## References


1 Kresse G, Furthmüller J. Efficient iterative schemes for ab initio total-energy calculations using a plane-wave basis set. *Physical Review B*, 1996, 54(16): 11169-11186.DOI:10.1103/PhysRevB.54.11169.

2 Dec Bo, Bogdanowicz R, Pyrchla K. Ab-initio study of electrical and optical properties of allylamine. *Photonics Letters of Poland*, 2018, 10(3): 94-96.DOI:10.4302/plp.v10i3.847.

3 Grimme S. Semiempirical gga-type density functional constructed with a long-range dispersion correction. *Journal of Computational Chemistry*, 2006, 27(15): 1787-1799.DOI:https://doi.org/10.1002/jcc.20495.

4 Sipper M, Moore JH. Conservation machine learning. *BioData Mining*, 2020, 13(1): 9.DOI:10.1186/s13040-020-00220-z.

5 Jia X, Li H. Machine learning enabled exploration of multicomponent metal oxides for catalyzing oxygen reduction in alkaline media. *Journal of Materials Chemistry A*, 2024, 12(21): 12487-12500.DOI:10.1039/D4TA01884B.

6 Christoph Höner zu Siederdissen NKB, Markus Cornberg. Reply to liaw. *The Journal of Infectious Diseases*, 2018, 218(11): 1853-1854.DOI:10.1093/infdis/jiy396.

7 Fereydooni K, Nordness O. Predicting the properties of il-solvent electrolytes using a hybrid support vector classification and gradient boosting regression (svc-gbr) framework. *Industrial & Engineering Chemistry Research*, 2025, 64(39): 19281-19294.DOI:10.1021/acs.iecr.5c02503.

8 Bartlett DJ, Desmond H, Ferreira PG. Exhaustive symbolic regression. *IEEE Transactions on Evolutionary Computation*, 2024, 28(4): 950-964.DOI:10.1109/TEVC.2023.3280250.

9 Xu Y, Qian Q. I-sisso: Mutual information-based improved sure independent screening and sparsifying operator algorithm. *Engineering Applications of Artificial Intelligence*, 2022, 116(105442.DOI:https://doi.org/10.1016/j.engappai.2022.105442.
